# Exosome secretion affects social motility in *Trypanosoma brucei*

**DOI:** 10.1371/journal.ppat.1006245

**Published:** 2017-03-03

**Authors:** Dror Eliaz, Sriram Kannan, Hadassa Shaked, Gil Arvatz, Itai Dov Tkacz, Lior Binder, Hiba Waldman Ben-Asher, Uthman Okalang, Vaibhav Chikne, Smadar Cohen-Chalamish, Shulamit Michaeli

**Affiliations:** The Mina and Everard Goodman Faculty of Life Sciences and Advanced Materials and Nanotechnology Institute, Bar-Ilan University, Ramat-Gan Israel; University of California, Los Angeles, UNITED STATES

## Abstract

Extracellular vesicles (EV) secreted by pathogens function in a variety of biological processes. Here, we demonstrate that in the protozoan parasite *Trypanosoma brucei*, exosome secretion is induced by stress that affects *trans*-splicing. Following perturbations in biogenesis of spliced leader RNA, which donates its spliced leader (SL) exon to all mRNAs, or after heat-shock, the SL RNA is exported to the cytoplasm and forms distinct granules, which are then secreted by exosomes. The exosomes are formed in multivesicular bodies (MVB) utilizing the endosomal sorting complexes required for transport (ESCRT), through a mechanism similar to microRNA secretion in mammalian cells. Silencing of the ESCRT factor, *Vps36*, compromised exosome secretion but not the secretion of vesicles derived from nanotubes. The exosomes enter recipient trypanosome cells. Time-lapse microscopy demonstrated that cells secreting exosomes or purified intact exosomes affect social motility (SoMo). This study demonstrates that exosomes are delivered to trypanosome cells and can change their migration. Exosomes are used to transmit stress signals for communication between parasites.

## Author summary

Trypanosomes are the causative agent of major parasitic diseases such as African sleeping sickness, leishmaniosis and Chagas' disease that affect millions of people. These parasites cycle between an insect and a mammalian host. Communication between the parasites and the host must be essential for executing a productive infection and for cycling of the parasite between its hosts. Exosomes are 40-100nm vesicles of endocytic origin, and were shown to affect a variety of biological processes and human diseases. Exosomes were also shown to help pathogens evade the immune system. In this study, we demonstrate that exosomes are secreted from *Trypanosoma brucei* parasites when *trans*-splicing is inhibited. These exosomes contain, among many other constituents, a type of RNA known as spliced leader RNA (SL RNA), which is essential in these parasites for formation of all mature mRNA. These exosomes are able to enter neighboring trypanosomes, and only intact exosomes affect the social motility of these parasites. We propose that exosomes can potentially control parasite migration in the insect host by acting as a repellent that drives the fit parasites away from either damaged cells or an unfavorable environment. This mechanism could secure a productive infection.

## Introduction

The African trypanosomes are protozoan parasites that cause a fatal infection in both humans and livestock, and constitute a major economic burden. These parasites cycle between two hosts, mammals and flies. The parasites are transmitted to the mammalian host by the tsetse fly, which feeds on the blood of an infected mammal. The parasites differentiate into the procyclic form (PCF) in the midgut. After about 2–3 weeks in the midgut, a few parasites migrate to the salivary glands where they differentiate to epimastigotes and then to metacyclics. The metacyclics are transmitted to the mammalian host, and the parasite then propagates in the host as bloodstream form [1, 2]. The active migration of the parasites within the insect host is essential for moving to the midgut against active forces of the digestive flow as the parasites enter the fly, but is also essential for migration from the midgut via the proventriculus to the salivary glands. This migration constitute a major bottleneck in the parasite life cycle [3].

In trypanosomes, all mRNAs are *trans*-spliced, a process that involves the addition of a spliced leader (SL) sequence from a small RNA donor, designated the SL RNA [4, 5]. The SL RNA undergoes co-transcriptional specific modifications to form the cap-4 hypermodified structure [6, 7]. When SL RNA biogenesis is blocked in cells silenced for Sm proteins, SMN, or GEMIN2 factors [8–10], SL RNA accumulates first in the nucleus, and then migrates to the cytoplasm to form distinct cytoplasmic granules. In contrast, the levels of all Sm-bound snRNAs are reduced [9]. Thus, excess SL RNA accumulation, which results from perturbation of *trans*-splicing, may have a biological function. Recent studies support the notion that RNA molecules are used as vehicles for cellular signaling. For example, microRNAs secreted from cancer cells via exosomes affect gene expression of neighboring cells, thereby influencing the tumor microenvironment [11–13]. Exosomes may also deliver information important for cell to cell communication [12].

Exosomes are 40-100nm vesicles of endocytic origin that are formed within multivesicular bodies (MVBs). Exosomes are secreted from cells by fusion with the plasma membrane. This process involves the endosomal sorting complex, required for transport (ESCRT) [14, 15].

Exosomes were shown to affect a variety of biological processes and have been associated with progression of neurodegenerative diseases, cardiovascular diseases, and cancer [15]. For example, exosomes help pathogens evade the immune system [16, 17]. In another “escape” mechanism, *Leishmania* uses exosomes to deliver the GP63 protease to hepatic cells where it inhibits Dicer1, blocking the processing of miR-122, whose level affects parasite burden [18]. Studies in malaria demonstrate that exosome-like vesicles secreted from infected red-blood cells (RBC) are involved in cellular communication, and sense population density during infection, to regulate the balance between asexual growth and production of gametocytes [16, 19, 20]. Exosomes, as well as other extracellular vesicles produced during viral, parasitic and bacterial infections could function to either promote or inhibit host immunity [21].

Extracellular vesicles were shown to be secreted from *T*. *brucei* [22], but their biological role was not explored until recently [23]. Recent studies provided evidence that the *T*. *brucei* bloodstream form, which propagates in the mammalian host, produces nanotubes that originate from the flagellar membrane and dissociate to form extracellular vesicles (EVs). These vesicles contain several flagellar proteins that function as virulence factors, as well as serum-resistance associated protein (SRA), which is required to avoid human infectivity. The EVs can fuse to human erythrocytes *in vivo*, causing the erythrocytes to be cleared more rapidly from the circulation. It was further suggested that EVs may be used by trypanosomes to evade the innate immune response [23].

Social motility (SoMo) was reported in *T*. *brucei* PCF [24, 25]. SoMo was shown to be a feature of early PCF, which are present in the lumen midgut [25]. Cell density sensing was also reported in the bloodstream stage of the parasite [26]. When two cohorts of PCF parasites come in close proximity, the parasites change their direction to avoid contact, suggesting that the parasites react to a repellent secreted from the cells [3, 24, 25, 27, 28]. The nature of this repellent is unknown. A strain that is mutant in N-linked glycosylation was shown to be defective in either the production or the perception of a migration-stimulating factor. However, this mutant can still react to repelling signals, suggesting that the repellent and migration factors are distinct [29]. cAMP and adenylate cyclase were shown to regulate social motility, and decreased levels of cAMP in the cell favor SoMo [30, 31]. SoMo might be essential for the parasite to traverse the peritrophic matrix to the ectoperitrophic space en-route to the salivary glands. The advantage to the parasite of such a mechanism is not currently known [27, 28].

In this study, we demonstrate that exosomes are secreted from cells when *trans*-splicing is inhibited. Exosome secretion is mediated by the ESCRT machinery. Silencing of *Vps36*, an ESCRT component, compromised the secretion of exosomes, but not of nanotube derived EVs, indicating that the secretion of these two types of vesicles utilize different mechanisms. These two types of vesicles are biochemically distinct. Time-lapse microscopy demonstrates that cells secreting SL RNA-containing exosomes affect the social motility of the parasites. Intact purified exosomes enter the trypanosome cells and inhibit migration. We propose that exosomes can potentially control parasite migration in the insect host by acting as a repellent that drives the fit parasites away from either damaged cells or an unfavorable environment. This mechanism could secure a productive infection.

## Results

### Cytoplasmic SL RNA is bound by distinct subset of proteins

Studies from mammalian cells suggest that secreted RNA found in exosomes affects the microenvironment of cancer cells promoting their growth [32]. The finding that the nuclear SL RNA, especially in cells silenced for the SL RNA core proteins (Sm proteins), is exported to the cytoplasm instead of undergoing degradation [8–10], motivated us to understand this phenomenon and explore whether the secretion of cytoplasmic SL RNA has a biological function analogous to the role of exosomes in cancer.

**Fig 1:**
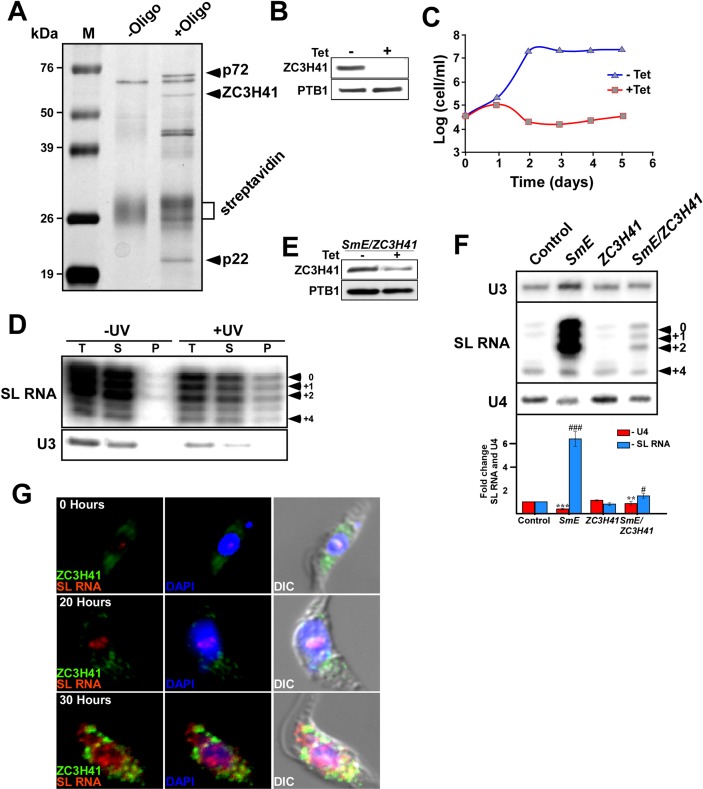
Identification of SL RNA associated proteins. **(A) Identification of proteins purified by affinity-selection**. Cells containing the *SmD1* silencing construct were induced for 48h, as previously described [9]. An extract was prepared from 2×10^9^ cells. The extract was separated on a Superdex 200 column, and SL RNA containing fractions were subjected to affinity selection as described in Materials and Methods. Proteins obtained from the control experiment lacking the selecting oligonucleotide (-Oligo) and proteins from the affinity selected particles (+Oligo) were extracted from the streptavidin beads, separated on a 12% acrylamide SDS gel, and stained with silver. **(B) *ZC3H41*silencing.** Cell lines expressing the *ZC3H41* stem-loop silencing construct were induced for 48 hrs. Cells (~10^6^ cells/ lane) were subjected to western analysis using ZC3H41 and PTB1 antibodies. **(C) *ZC3H41* is an essential gene for trypanosome survival.** Cells were either induced (+Tet) or un-induced (-Tet), and growth was monitored. The arrow indicates the time of tetracycline addition. The number of un-induced cells is designated by triangles, and of induced cells by squares. **(D) ZC3H41 binds loosely to SL RNA.** Cells expressing TAP-Myc-His- ZC3H41 fusion protein and the *SmD1*-silencing construct were silenced for 48 hrs. Cells (1.5×10^9^) were UV irradiated, as described in Materials and Methods. Extracts prepared from control (-UV) cells, and cells following UV irradiation were affinity selected on IgG beads. The RNA was extracted from the beads and analyzed by primer extension with SL and U3 RNAs specific primers. T, Total extract (5%); S, supernatant after removing the IgG beads (5%); P, the entire RNA sample bound to beads. The position of the cap-4 modification is indicated. **(E) Depletion of ZC3H41 in *SmE/ ZC3H41* silenced cells.** Western analysis was performed, as described in panel B. **(F) Levels of SL RNA under *SmE* and *SmE/ ZC3H41* silencing.** 10 μg of total RNA was subjected to primer extension with primers specific to SL RNA, U4, and U3 snoRNAs (S1 Table). The extension products were separated on a 6% denaturing gel. The identity of the cell line and the position of the modified cap nts are indicated. The statistical analysis represents the mean ± s.e.m of quantification from three independent experiments. **P <0.01, and ***P <0.005 compared to–Tet, using Student's *t*-test. **(G) Changes in localization of ZC3H41 and SL RNA during *SmD1* silencing.** Cells carrying the *SmD1* silencing construct were induced for the time indicated and subjected to *in situ* hybridization with SL RNA (red) and IFA with ZC3H41 antibodies (green). The nucleus was stained with DAPI. The merge was performed on DAPI staining and SL RNA hybridization and the time points of silencing are indicated.

To address this question, we sought to first identify the proteins that are bound to the SL RNA in the cytoplasm [8, 9]. To this end, we purified the SL RNA-associated proteins using gel filtration columns combined with affinity selection with an anti-sense 2’-*O*-methyl biotinylated oligonucleotide. The affinity selection used an oligonucleotide that was shown previously to specifically select the SL RNP [33]. Indeed, in our hands as well, the affinity selection was very specific, yielding a distinct subset of polypeptides that appear only after selection with the anti-sense oligonucleotide (Fig 1A, S1 Fig). Mass spectrometry (MS) of proteins bound to the beads lacking oligonucleotide (-oligo) did not detect any of the proteins identified in the affinity-selected sample (S2 Table). To identify the specific proteins that were selected with anti-sense biotinylated oligonucleotide, prominent polypeptides were excised and analyzed by mass spectrometry. The purifications were repeated three times. Several proteins, especially p58, p22, and p72, were identified (marked on Fig 1A). The identity of the proteins was determined. p22 is a disulfide isomerase, and p72 belongs to the ATPase family of the ABC transporters (S2 Fig); p58 was found to have interesting functional domains associated with RNA metabolism, such as helicase C, zinc finger CCCH, a sterile alpha motif (SAM), as well as a KH domain (S2 Fig). This protein was recently identified in stress granules and was termed ZC3H41 [34]. The protein also has partial similarities to DHH1 and to the eIF4ΑI and III helicases, mostly in the helicase domain. Because of its potential involvement in RNA-metabolism, we chose ZC3H41 as the marker for SL RNA containing granules throughout this study.

Mass-spectrometry was performed for identification of all the proteins affinity-selected with SL RNA in three separate purifications (S2 Table). Note, that the proteins discussed above were among the most abundant proteins in the three purifications. However, differences were observed between the different purifications, possibly because each purification may have captured the complex at different stages of its biogenesis. Silencing performed on a large sample of cells (needed for the purification) can vary. The cytoplasmic SL RNA complex might be dynamic in its composition from the moment the SL RNA is translocated from the nucleus until its assembly in exosomes (see below). The MS data obtained from the three biological replicates was unexpected, since these proteins were not shown previously to associate with SL RNA [33, 35], and except for ZC3H41, the function of these proteins is not known from other studies. However, we were able to consistently purify these proteins. Note, that none of these proteins appeared in the MS of the (-oligo) control. To investigate whether the identified proteins (Fig 1A, S2 Table) are indeed genuine constituents of this complex, the effect of their depletion on formation/stability of the complex was examined. *ZC3H41* was silenced by RNAi using a stem-loop construct [36]. Antibodies were raised against the protein, and were used to verify the depletion in the silenced cells (Fig 1B). The gene was found to be essential for growth (Fig 1C). Next, the direct association of SL RNA with ZC3H41 was examined by affinity selection of the SL RNA with ZC3H41-TAP tagged protein. Since the cytoplasmic SL RNA complex was found to be fragile, the association of the SL RNA with ZC3H41 was examined following UV cross-linking. Whole cell extracts was prepared from the cells carrying the *SmD1* silencing construct and expressing the ZC3H41 TAP-tagged protein. The cells were either cross-linked with UV or not irradiated (control), and the selected RNA was subjected to primer extension. SL RNA was selected only following UV irradiation. The specificity of the selection was evident from the U3 control (Fig 1D). Although the association appeared to be specific and higher than in the control, it was not very strong, and it is possible that the association does not occur through direct RNA binding, but rather that ZC3H41 is among the proteins that are associated with the SL RNA in granules.

The role of ZC3H41 in the accumulation of SL RNA was further probed by silencing of *ZC3H41* together with *SmE*. The double-silencing resulted in considerable reduction in the level of ZC3H41 (Fig 1E), and a significant reduction in the amount of SL RNA that accumulated in these silenced cells (only 1.8-fold elevation) compared to 7.4-fold elevation under *SmE* silencing, suggesting that ZC3H41 is essential for the accumulation of SL RNA (Fig 1F). Note that silencing of *SmE* and *SmD1* result in the same phenotype [9]; these silenced cell lines were used interchangeably in this study, since these proteins together constitute the SL RNA core Sm complex [37]. Double silencing of *SmE/ ZC3H41* was less efficient than silencing of *ZC3H41* alone, because double silencing requires the silencing machinery to silence two genes instead of one, and the silencing machinery might be exhausted. Despite the somewhat diminished silencing efficiency, the U4 snRNA level was reduced in these cells, indicating the efficient silencing of the Sm protein (Fig 1F). Similar analyses were performed for the additional SL RNA-associated proteins, p22 and p72 (S3 Fig). Depletion of these proteins reduced the level of SL RNA accumulation, but reduction of U4 snRNA was observed, verifying the efficiency of *Sm* knock-down (S3 Fig). All the SL RNA-associated proteins were found in the cytoplasm, and formed granules under *SmE* silencing (S3 Fig). Thus, these results demonstrate that all the proteins which were consistently purified with cytoplasmic SL RNA are essential for the accumulation of SL RNA and granule formation.

Immunofluorescence staining with ZC3H41 antibodies coupled with *in situ* hybridization with SL RNA indicated that under normal conditions, ZC3H41 was localized near the nucleus, and SL RNA was found within the nucleus (Fig 1G, S4 Fig). However, following *SmD1* silencing, ZC3H41 formed cytoplasmic granules that appeared even before the majority of SL RNA migrated to the cytoplasm (20 hrs silencing). Only partial co-localization was observed with the cytoplasmic SL RNA (30 hr silencing), suggesting that ZC3H41 may have additional function(s) in these silenced cells apart from its association in the SL RNA granules. Indeed, a recent study identified ZC3H41 among the proteins present in stress granules induced under serum starvation [34]. Thus, two types of granules may exist in these *SmD1*-silenced cells, SL RNA granules and stress granules (see below). The mechanism by which the SL RNA is translocated from the nucleus to the cytoplasm and how SL RNA is assembled with the proteins identified here, is currently unknown.

### SL RNA granules are distinct from P-bodies and stress granules

**Fig 2:**
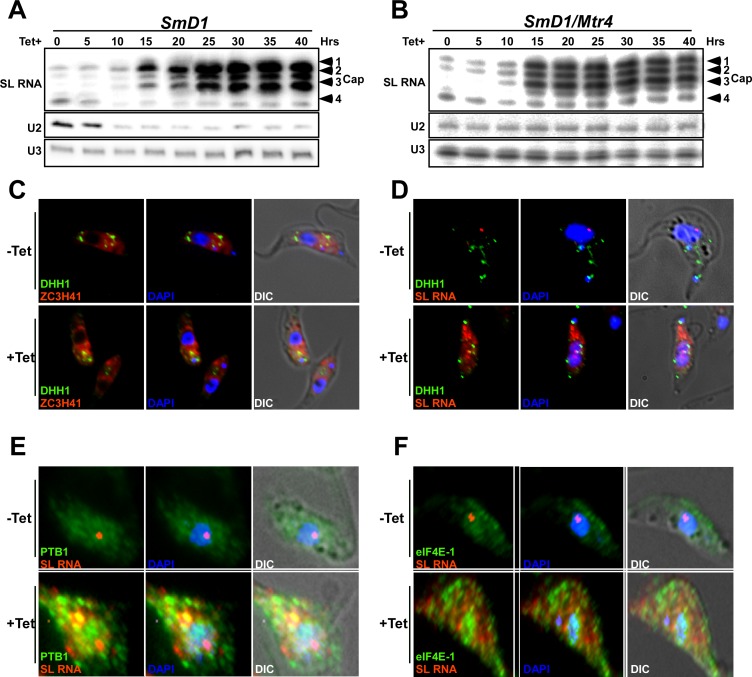
MTR4 regulates the level of U snRNA, but does not affect SL RNA, under *SmD1* silencing. **(A) Primer extension of SL, U2 and U3 RNAs during *SmD1* silencing.** Total RNA (10 μg) was extracted from cells silenced for the indicated times, and subjected to primer extension. The extension products were separated on a 6% denaturing gel. **(B)** The same experiment as in **(A)** but using RNA extracted from *SmD1/Mtr4* silenced cells. **(C) SL RNA granules are not P bodie**s. ZC3H41 localization with respect to P-bodies labeled with DHH1. Cells carrying the *SmD1* silencing construct, and the YFP-DHH1 construct were silenced for 40 hrs. and stained for IFA using ZC3H41 and YFP antibodies (red and green respectively). The nucleus was stained with DAPI. **(D) *In situ* hybridization with SL RNA.** Cells carrying the *SmD1* silencing construct and expressing YFP-DHH1 were induced for 40 hrs. and subjected to *in situ* hybridization with SL RNA (red) and immunofluorescence using YFP antibody for YFP-DHH1 (green). The nucleus was stained with DAPI. **(E) SL RNA granules are not stress granules**. Cells were silenced for 40 hrs. and stained by IFA using PTB1 antibodies (green stain) and were subjected to *in situ* hybridization with SL RNA (red). The nucleus was stained with DAPI. **(F)** as in **(E)** but using antibodies to eIF4E-1. The merge was performed between DAPI staining, IFA and *in situ* hybridization.

One of the puzzling questions regarding the massive accumulation of SL RNA is why the defective SL RNA is not degraded in the nucleus, but is transported to the cytoplasm. To address this issue, the role of the nuclear surveillance mechanism on SL RNA homeostasis was examined. The nuclear helicase, MTR4, controls the steady-state levels of U snRNA and recruits the machinery that degrades defective non-coding RNAs [38, 39]. To examine the possibility that MTR4 regulation operates in trypanosomes to degrade SL RNA, in a manner analogous to snRNA in other eukaryotes [38, 39] the effect of *Mtr4* silencing on these RNAs was examined (S5A Fig). Whereas silencing of *SmD1* resulted in reduction of U2 snRNA and elevation of SL RNA, *Mtr4* silencing abrogated U2 snRNA degradation, while SL RNA levels were not affected (compare Fig 2A to Fig 2B). The quantification of this phenomenon is presented (S5B and S5C Fig) indicating that the changes in the level of SL RNA occurred with similar kinetics as that of the U2 snRNA, suggesting that depletion of Sm proteins had a direct effect on both RNAs. These results also demonstrate that SL RNA is resistant to degradation by the nuclear surveillance mechanism. This phenotype may stem from overwhelming the surveillance machinery at the SL RNA transcription and assembly site, which is different from the site of snRNA assembly with Sm proteins [37]. Note also that when SL RNA is exported to the cytoplasm, it is no longer under the control of the nuclear exosome, and cannot be degraded, and hence accumulates.

Next, we examined whether SL RNA granules are related to P-bodies or stress granules, which are also known in trypanosomes to store factors involved in RNA metabolism [34, 40, 41]. A fraction of ZC3H41 was found in P-bodies (labelled by YFP-DHH1), but the majority of this protein was found outside P-bodies (Fig 2C, S5D Fig). Most importantly, SL RNA was also absent from P-bodies (Fig 2D, S5E Fig). Thus, although ZC3H41 is found in P-bodies, SL RNA is not, indicating that the SL RNA granules are distinct from P-bodies. Next, we examined co-localization of SL RNA with stress granules and investigated the distribution of two proteins that were identified in stress granules, PTB1 and eIF4E-1 [34]. PTB1 regulates splicing but also functions to control mRNA decay [42], and eIF4E-1 was shown in *Leishmania* to function during thermal stress [43]. These proteins formed granules under *SmD1* silencing, but these granules did not contain SL RNA, suggesting that SL RNA granules are not stress granules but rather represent a distinct inclusion. However, stress granule formation is induced under *SmD1* silencing (Fig 2E and 2F). Additional images describing the relationship between DHH1, PTB1, eIF4E-1 and SL RNA are presented in (S5E–S5G Fig) supporting the finding that SL RNA-containing granules are distinct from P-bodies and stress granules.

Next, we followed the fate of cytoplasmic SL RNA. Based on the finding that secretion of microRNAs from cancer cells is mediated by exosomes [11–13], we examined whether the *SmD1* silenced cells secrete exosomes harboring SL RNA.

**Fig 3:**
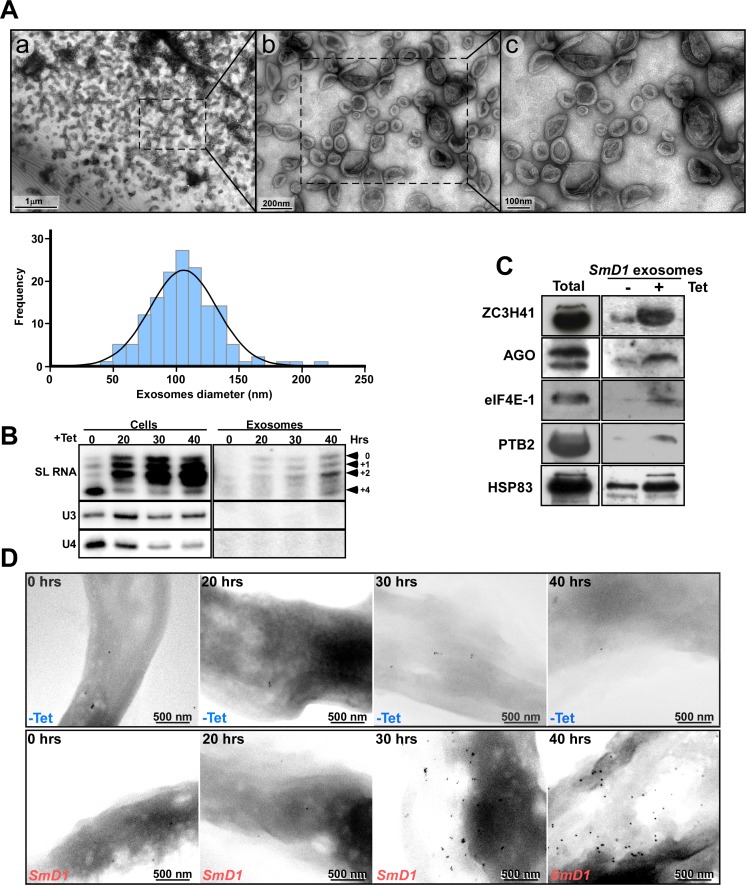
Exosomes secreted from *SmD1* silenced cells. **(A) TEM analysis of the exosome-enriched fraction from *SmD1* silenced cells.** Exosomes were purified, from 10^9^ cells after 40 hrs of silencing, and were subjected to TEM. **(a)** The entire field of exosomes; **(b)** and **(c)** show enlargement of a section indicated by a square on panel **(a). Exosome size distribution.** For the analysis, the size of 150 exosomes was determined from ten different images using ‘NIS-elements’ imaging Software (Nikon, USA). The data were analyzed by the one-sample Kolmogorov-Smirnov test (SPSS IBM, USA). The average size of each exosome was 106.1 ± 26.6 nm (p value for normal distribution, 0.69). **(B) RNA is secreted via exosomes.** Cells containing the *SmD1* silencing construct were silenced for the indicated times (hours). RNA was prepared from the cells and from exosomes as described in Materials and Methods. The RNA was subjected to primer extension. The products were separated on a 6% acrylamide denaturing gel. The cap-4 nts are indicated. **(C) Proteins are secreted with the exosomes.** Proteins were extracted from exosomes prepared from 10^9^ cells and were subjected to western analysis with the indicated antibodies. Total cell extract (20 μg) was used to monitor the specificity of the antibodies. **(D) Immunogold-SEM detecting ZC3H41 protein on the surface of the parasite.** Un-induced cells containing the *SmD1* silencing construct (-Tet) and *SmD1* silenced cells at the indicated time points were subjected to immunogold staining with specific anti-ZC3H41 antibodies. Backscatter images are presented; scale bars are indicated.

Purified exosomes were analyzed by TEM (Fig 3A). A section from the field presented in (Fig 3Aa) was enlarged (panels b and c) revealing the bilayer bounding membrane of the exosomes. The exosomes observed are similar to those described in *Leishmania* and trypanosomes [22, 44–47] ranging in diameter from 50-to 200 nm, with an average size of 100 nm (Fig 3A). Note, that the cup shaped nature of the exosomes may be an artifact resulting from the use of vacuum during the specimen preparation.

Next, we examined whether SL RNA is secreted by exosomes. Indeed, SL RNA was preferentially secreted, whereas no U3 snoRNA was detected (Fig 3B). The secretion of proteins via exosomes was next examined (Fig 3C). Secretion of the indicated proteins was observed, along with Argonaute (AGO) and HSP83, which are known to be secreted by the exosomes [48–50]. Since stress granules are formed along with SL RNA-containing granules in *SmD1* silenced cells, it was important to determine whether proteins associated with stress granules are also secreted via exosomes. Our results indicate that stress granule proteins such as PTB2 and eIF4E-1 were barely detected in these exosomes. On the other hand, ZC3H41 was found to be massively secreted via these exosomes, and this protein was therefore chosen as a marker for exosomes secreting SL RNA. To further study exosome secretion, it was important to monitor the optimal time point for studying the process. To this end, SEM analysis combined with immunogold staining using antibodies to ZC3H41 (Fig 3D) demonstrated that secretion of ZC3H41 from *SmD1* starts ~30 hrs. post induction, and secretion increases with time over the next few days. Based on these results, we decided to study the secretion process and its biological effects at least ~ 40 hrs. post silencing induction.

### The secretion of exosomes from *SmD1* silenced cells is mediated by MVBs and requires ESCRT function

The recent report of EV secretion from trypanosomes demonstrated that these emerge from the flagellar membrane [23]. In higher eukaryotes, exosomes are formed in the MVB, which are defined as late endosomes that contain intraluminal vesicles (ILV). MVB formation is mediated by ESCRT 0-IV complexes, which are responsible for loading the cargo, initiate vesicle invagination, and induce internal vesicle membrane scission [15]. Factors involved in MVB biogenesis were shown to exist in trypanosomes, including ESCRT complex functions [51, 52].

**Fig 4:**
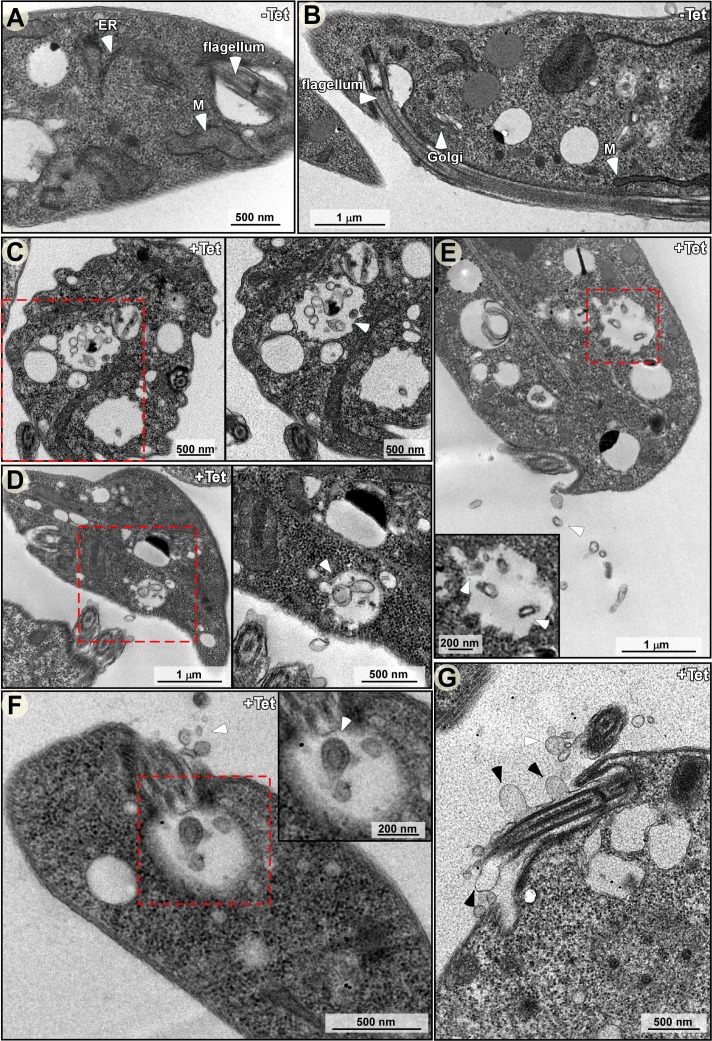
TEM analysis of *SmD1* silenced cells. Cells before and after silencing of *SmD1* (40 hrs) were fixed, and ultra-thin sections were prepared and examined by TEM. The different ultra-structures are indicated. MVB, multivesicular bodies; ILV, intraluminal vesicles; M, mitochondrion; ER, Enodoplasmic reticulum; scale bar, 500 nm. (**A and B**) Un-induced cells (-Tet). (**C-G**) induced cells. Section showing MVB containing ILVs. **(C-D)** MVB located near the plasma membrane, and secreted exosome present on the cell surface. **(E-F)** EVs secreted from the cell membrane and flagella pocket. **(G)** Larger vesicles emerging from the flagellar membrane. The white arrow-heads indicate the ILVs and black arrow-heads the EVs.

To examine whether MVB-type structures exist in trypanosomes, and whether these contain ILVs, cryo-TEM sections were prepared from cells before (Fig 4A and 4B) and after silencing of *SmD1*. MVBs carrying ILVs were observed in the silenced cells (boxed in Fig 4C to 4F). Each panel (4C-4F) is presented at two magnifications.

The results reveal two types of vesicles. Small vesicles were found mainly inside MVBs (Fig 4C to 4F). Vesicles of similar size were also secreted from the flagellar pocket (Fig 4F). In addition, larger vesicles also emerged from the flagellar membrane (marked with an arrow) (Fig 4G), much like the EVs described recently in bloodstream trypanosomes [23]. However, the two types of vesicles, EVs and exosomes, seemed to differ in size.

The formation of a large number of MVBs containing ILVs was specific to the *SmD1* silenced cells. Inspecting TEM sections of *SEC63* silenced cells, which induce the spliced leader silencing (SLS) pathway [53], revealed the presence of another type of vesicle, double-membrane autophagosomes [54], but not MVBs filled with ILVs, as in *SmD1* silenced cells (S6 Fig); thus MVBs seem to be specific to cells secreting massive amounts of exosomes. Small numbers of exosomes are secreted from *SEC63* silenced cells (S7 Fig).

**Fig 5:**
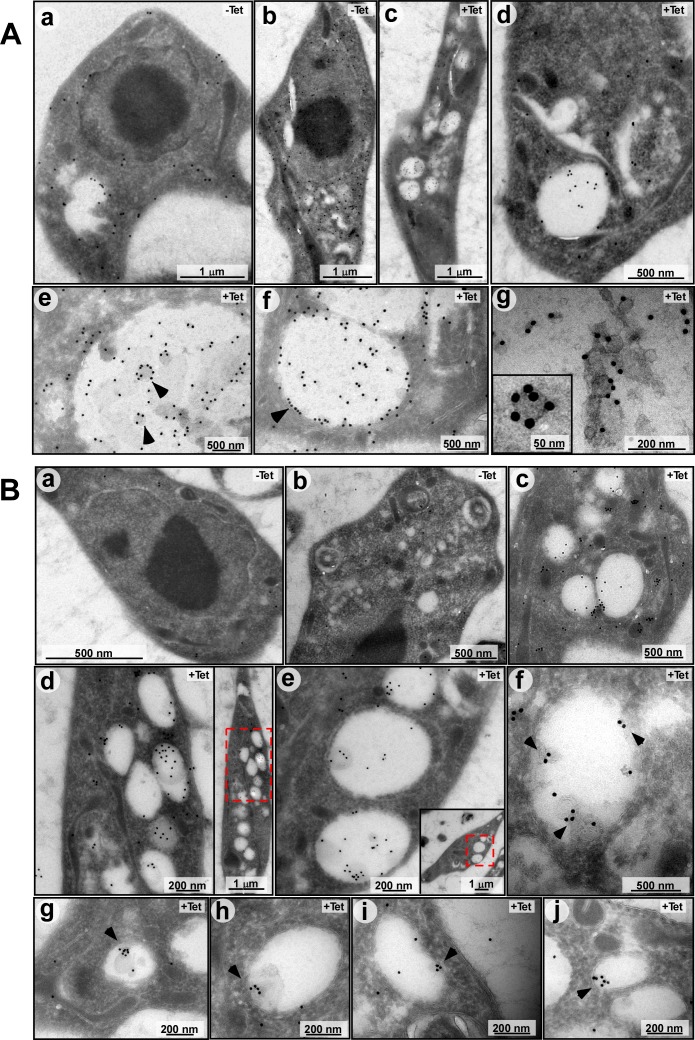
**(A) Immunogold TEM analysis demonstrating the location ZC3H41 in ILVs within MVBs.** Cells carrying the *SmD1* silencing construct, and the YFP-VPS36 construct were used. **(a, b)** un-induced (-Tet) or **(c-g)** silenced for 40 hrs. (+Tet) were used to prepare Cryo-EM sections, and were subjected to immunogold analysis. Imaging was performed using antibody to ZC3H41. **(B)** as in **(A)**, but using anti-GFP antibody to detect the VPS36. The scale bars are indicated.

To verify that these ILVs are the source of exosomes, the cryo-EM sections were probed for the presence of ZC3H41 or VPS36 proteins using immunogold microscopy. VPS36 was chosen because its depletion in mammalian cells affects exosomal secretion of microRNAs [11, 12]. Localization of ZC3H41 in un-induced cells was mostly in the cytoplasm (Fig 5Aa, b) Following *SmD1* silencing, ZC3H41 was observed mostly in the ILVs (Fig 5Ac-f). The high magnification images (Fig 5Ag) show that ZC3H41 is found mostly at the surface of the vesicle. Next, the ILVs were visualized using YFP-tagged VPS36. MVB containing ILVs were not observed in un-induced cells (Fig 5Ba,b), while ILVs containing VPS36 were observed in the silenced cells (Fig 5Bc-j). These results suggest that as a result of *SmD1* silencing, MVBs containing ILVs are formed. As in other eukaryotes, these ILVs are the source of exosomes.

**Fig 6:**
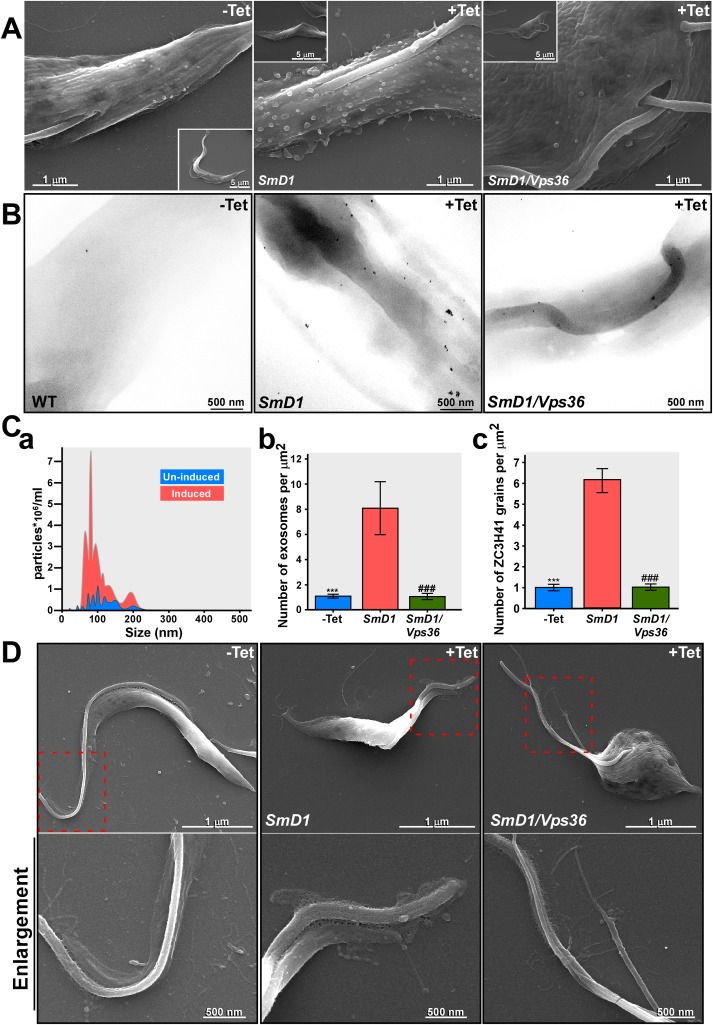
Exosome detection by SEM of *SmD1* and *SmD1/Vps36* silenced cells. **(A)** Cells carrying the *SmD1* silencing construct before (-Tet), and after 40 hrs of silencing (+Tet) were fixed and visualized under EM. The scale bars and the cells identity are indicated. **(B) SEM immunogold.** *SmD1* and *SmD1/Vps36* silenced cells as describe in A were subjected to immunogold analysis with ZC3H41 antibodies. The scale bars and the cells identity are indicated. **(C) Quantitative analysis of secreted exosomes (a)** NanoSight analysis. To quantitate the number of exosomes, exosomes were prepared from un-induced cells (10^9^) or after 40 hrs. of induction. The exosomes were analyzed by NanoSight. Un-induced (blue); *SmD1* silenced cells (red). (**b)** Quantitation of exosomes from EM images. The number of exosomes was determined by counting exosomes present in a given surface area. 12 independent images for each of the panels were analyzed. The number of exosomes per μm^2^ is given. Statistical analysis was performed using one-way ANOVA, post hoc–Bonferroni-test implemented in SPSS (IBM, USA) ***/^###^ *P* <0.005 compared to +Tet. **(c)** Quantitation of immunogold analysis with ZC3H41 antibodies. Twenty different images were analyzed. The size of each cell was calculated and the number of grains per μm^2^ is given. Statistical analysis was performed as described in panel **(Cb)**. **(D) High resolution SEM demonstrating nanotubes near the flagellar pocket.** Cells (as indicated on the top of each frame) before silencing (-Tet), and after 40 hrs of silencing were visualized by SEM. Higher magnification of the sections is indicated. The scale bars and the cells identity are indicated.

ESCRT factors were shown to be involved in RNA secretion, and silencing of *Vps36* compromises RNAi silencing in mammalian cells [55]. To test whether the ESCRT machinery is involved in SL RNA secretion, *Vps36* was silenced along with *SmD1*. Elimination of the *Vps36* did not interfere with SL RNA accumulation (S8A–S8C Fig). Perturbation of exosome secretion was clearly observed by SEM (Fig 6A), further demonstrating that silencing of *Vps36* compromised exosome secretion. Few if any exosomes were observed in cells co-silenced for *SmD1/Vps36*. The efficient silencing of *SmD1* was evident from the morphological changes in *SmD1* silenced cells, as clearly manifested by the altered shape of the entire parasite (insets in panel A). To quantitate the exosomes, secreted exosomes were prepared from unmanipulated cells, and from the same number of cells after 2 days of silencing. The exosomes were analyzed by NanoSight instrument which measures particles based on their light scattering. The results (Fig 6Ca) indicate a strong distinct peak corresponding to the size of the exosomes (~100 nm). Seven fold more exosomes were present in the preparation of exosomes from the silenced cells compared to control. The EM images (Fig 6A) were also used for quantitative measurement to determine exosome secretion. Exosomes were counted from several different images and their number is given per μm^2^ (Fig 6Cb). The EM quantitation indicated that ~8 fold more exosomes were present on the surface of silenced cells, compared to negligible numbers present on un-induced cells or in cells silenced for *SmD1/Vps36* (Fig 6Cb). Finally, since secreted exosomal proteins can be visualized by SEM immunogold using ZC3H41 antibodies, the gold particles can also be used as a measure of secretion. The number of immunogold grains was counted on the surface of the parasites (Fig 6B) and using this approach, ~6 fold more ZC3H41 grains (Fig 6Cc) were present on the surface of the *SmD1* silenced cells compared to un-induced cells or on the surface of *SmD1/Vps36* silenced cells.

A recent study in *T*. *brucei* described the budding of EVs from nanotubes [23]. It was therefore of great interest to reveal such nanotubes in procyclic-stage parasites. We observed nanotubes in the vicinity of the flagella (Fig 6D). Such nanotubes were clearly observed in the *SmD1/Vps36* silenced cells, suggesting that there are at least two distinct secretion pathways in trypanosomes, via MVBs and from the nanotubes. These results strongly indicate that nanotube-mediated secretion exists in procyclic stage trypanosomes, and thus, these nanotubes may have different functions in these two life stages of the parasite. However, EV secretion from nanotubes is not mediated by the ESCRT machinery.

### The SL RNA vesicles are biochemically distinct from stress granules

**Fig 7:**
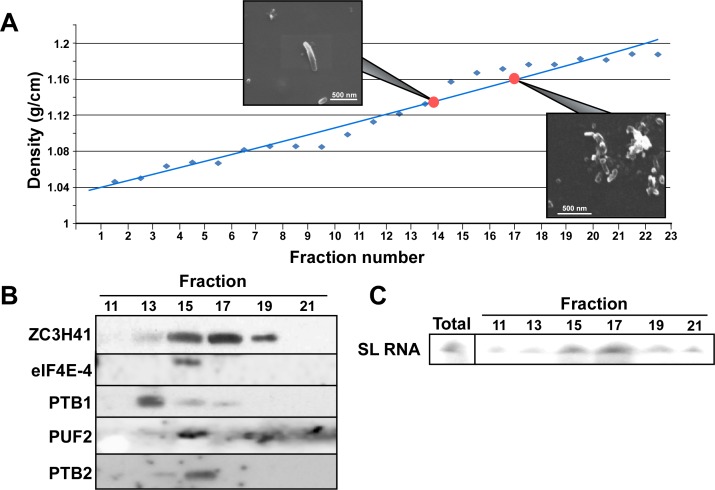
The SL RNA exosomes are distinct from stress granules and nanotubes. Exosomes were prepared from *SmD1*-silenced cells grown in medium containing FCS free of bovine exosomes, after 2 days of silencing. The exosomes were fractionated on a 15–50% sucrose gradient at 39,900 rpm in a SW41 rotor for 20 hr. Fractions (500 μl) were subjected to SEM and western analyses as well as to primer extension to monitor SL RNA. **(A) Fractionation of vesicles on the sucrose gradient.** The density of the fractions are given and SEM images derived from fractions 13–14 and fractions 17–18 are presented. **(B) Western analysis.** Fractions 10–21 were subjected to western analysis using the indicated antibodies. **(C) The location of SL RNA**. RNA was extracted from the different fractions and subjected to primer extension.

Since the *SmD1* silenced cells contain SL RNA and stress granules and these cells also secrete different vesicles, especially the previously described EVs [23], we sought to determine whether these are distinct entities that can be biochemically separated. To this end, exosomes were prepared from *SmD1* silenced cells and loaded onto 15–50% sucrose gradients. After centrifugation, the fractions were analyzed by SEM (Fig 7A). Proteins were subjected to western blot analysis and to primer extension with SL RNA. The results (Fig 7B) demonstrate that the nanotube fraction (density ~1.12mg/ml, fraction # 13) can be separated from the vesicles containing SL RNA and ZC3H41 (density 1.16–1.18, fraction # 17). Stress granule proteins fractionated at densities of 1.12–1.14 g/ml, but we could hardly detect exosomes in these fractions. These results suggest that under *SmD1* silencing, the most abundant secreted vesicles are those containing SL RNA and ZC3H41 (Fig 7C).

### SL RNA secretion via exosomes can be induced by heat-shock

**Fig 8:**
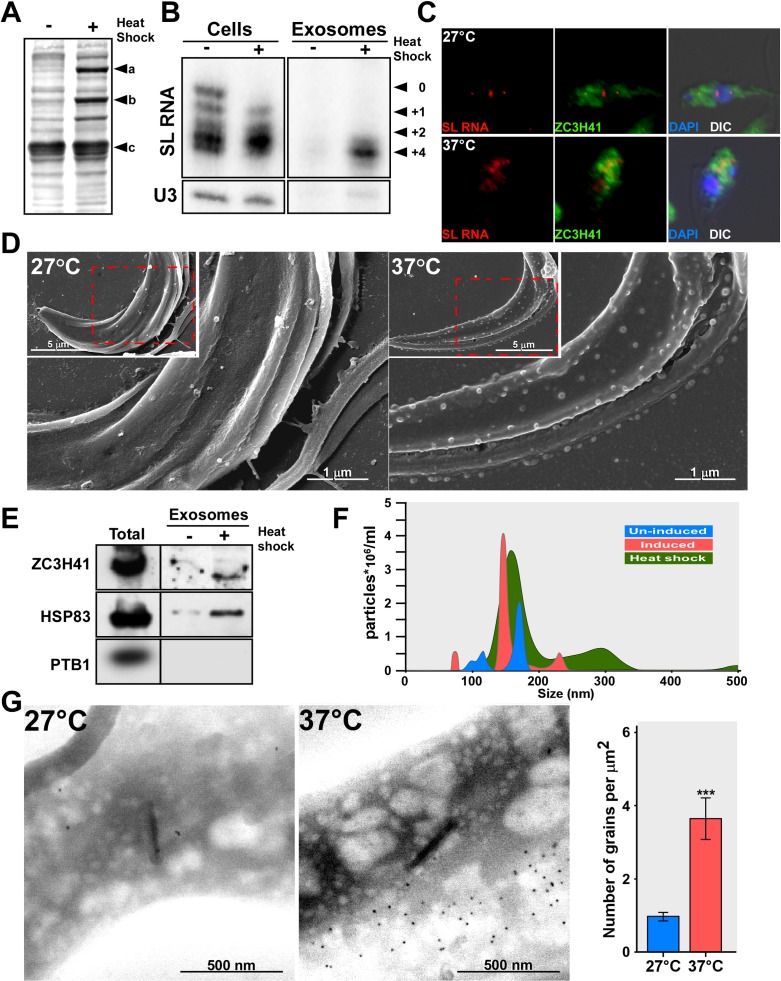
SL RNA accumulates during heat-shock and is secreted by exosomes. **(A) Induction of heat-shock proteins.** Cells (10^7^), were incubated at either 27°C or 37°C (heat-shock) for 40 minutes in Methionine-free medium; ^35^S-Methionine (100 μCi) was added for 10 min followed by a 5 min chase. The cells were collected, and the proteins were separated on a 10%-SDS gel and subjected to autoradiography.**a-HSP83 b-HSP70 c-tubulin. (B) SL RNA accumulates under heat-shock.** Cells (10^8^ in 10 ml) were incubated at 27°C or 37°C, for 1h. RNA was prepared from semi-purified exosomes and analyzed by primer extension. The SL RNA cap-4 modifications are indicated. **(C) SL RNA and ZC3H41 are transported from the nucleus to the cytoplasm under heat-shock.** Cells were incubated for 1hr at the temperature indicated and subjected to *in situ* hybridization. Nuclei were stained with DAPI. The merge was performed between IFA, *in situ* hybridization and DAPI staining. **(D) SEM analysis of un-induced cells, and cells exposed to heat-shock.** Cells were either incubated at 27°C or 37°C (heat-shock) for 1 hour. After incubation, the cells were fixed and visualized under EM; the scale bars and the treatment of the cells are indicated. **(E) Exosomes are secreted under heat-shock**. Cells (10^8^) were incubated at either 27°C or 37°C (heat-shock) for 40 min, and exosomes were prepared as described in Materials and Methods, and subjected to western analysis with the indicated antibodies. **(F) Quantitation of the exosomes secreted under heat-shock.** Exosomes from wild-type, *SmD1* silenced cells and heat-shocked cells were analyzed by NanoSight. Exosomes from un-induced cells (blue), *SmD1* silenced cells (red), and heat-shock (green). **(G) SEM-Immunogold to detect ZC3H41 secretion under heat-shock.** Wild-type cells were incubated for 1h at the indicated temperature. Cells were subjected to immunogold staining. The backscatter images are presented; scale bars are indicated. The statistical analysis represents the mean ± S.E.M **P <0.01, and ***P <0.005 compared to–Tet, using Student's *t*-test.

As *Sm* silencing is an artificial cue, we searched for a physiological phenomenon that also affects *trans*-splicing and SL RNA biogenesis, to examine if under such conditions, SL RNA might also be secreted by exosomes. Heat-shock causes defects in mRNA maturation in *T*. *brucei* [56], due to increased degradation, reduced transcription, and inhibition of *trans-*splicing [40]. Indeed, when *T*. *brucei* PCF cells were subjected to heat-shock at 37°C, expression of heat-shock proteins was induced (Fig 8A), and the cells remained viable under these conditions (S9 Fig). RNA levels were monitored upon heat-shock, and an increase in SL RNA was observed, possibly due to reduced *trans*-splicing (Fig 8B). Exosomes prepared from heat-shocked cells but not from cells grown at 27°C contained SL RNA (Fig 8B). Heat-shock inhibits *trans*-splicing, leading to the accumulation of SL RNA. However, heat-shock does not affect capping of SL RNA like Sm depletion. The capping defect found under Sm silencing results from the linkage between +4 cap modification and Sm assembly [9]. *In situ* hybridization combined with immunofluorescence (IFA) using antibodies to ZC3H41 demonstrated that under heat-shock, SL RNA was exported to the cytoplasm and was found in granules, as under *Sm* silencing (Fig 8C). Exosomes were visualized by SEM, and massive secretion was found on cells exposed to 37°C (Fig 8D). Exosomes prepared from heat-shocked cells were also enriched with ZC3H41 and HSP83, but not with PTB1 (Fig 8E). The amount of exosomes secreted for cells under heat-shock was monitored by NanoSight. The results (Fig 8F) suggest massive secretion of exosomes under heat-shock compared to cells under normal growth conditions. The secretion of ZC3H41 was also confirmed using immunogold (Fig 8G). Thus, exosome secretion takes place during heat-shock, and may occur when the fly host is exposed to high temperatures in the summer.

### Exosomes secreted from *SmD1* silenced cells enter trypanosomes

**Fig 9:**
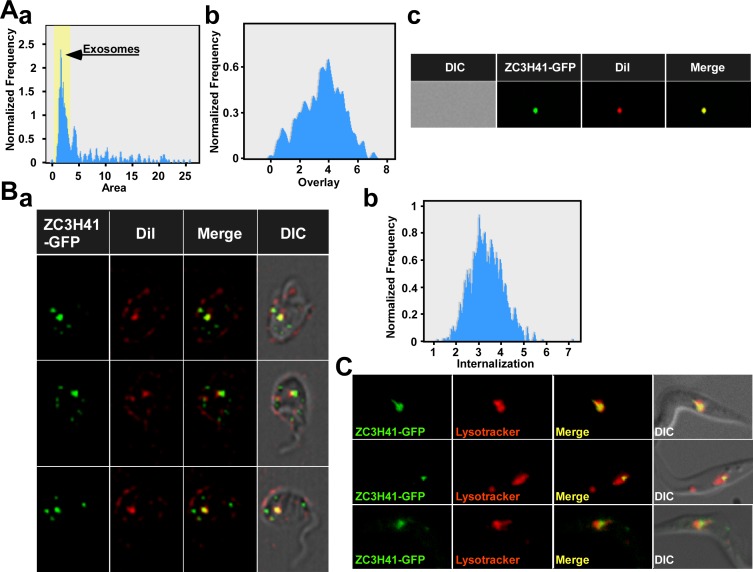
Entry of exosomes to target cells. **(A) Fluorescence of exosomes stained with DiL and containing the ZC3H41-GFP.** Exosomes (20μl, representing 1/10 of the exosome preparation from 10^9^ cells) were incubated at room-temperature with 1:1000 dilution of the DiL and were analyzed by ImageStream. **(a)** The number of particles versus their area is presented. The peak corresponding to exosomes is indicated. **(b) The overlap between the two fluorescent dyes. (c)** Single exosome fluorescence. The fluorescence of GFP and DiL was recorded in addition to the overlay. DIC, and the different fluorophores are indicated. **(B) Exosomes enter wild-type trypanosome cells. (a)** The exosomes described in **(A)** (40 μl) were incubated with 100μl containing 10^6^ cells. ImageStream analysis was performed after 10 minutes, and single-cell fluorescence of ZC3H41–GFP and DiL was monitored. **(b)** The level of internalization of the two fluorophores. **(C) Live imaging of cells incubated with ZC3H41-GFP exosomes and lysotracker.** Exosomes were prepared from 10^9^ cells, and 1/10 of the preparation (20 μl) was incubated with 100μl containing 10^6^ cells for 1 hr and then lysotracker was added, and cells were visualized.

For the exosome to exert any biological activity on neighboring parasites they must enter to recipient cells. To monitor the entry of exosomes to trypanosomes, exosomes were prepared from cells expressing ZC3H41–GFP and the *SmD1* silencing construct. Exosomes were purified from silenced cells and were mixed with the lipophilic dye, carbocyanine DiL, which stains the exosome membrane. The exosomes were analyzed by ImageStream, and a single major peak was observed (Fig 9Aa). The peak was analyzed, and the results indicate significant overlap between the two fluorophores, suggesting that the exosomes were efficiently labelled by DiL (Fig 9Ab). Indeed, fluorescence within a single exosome shows co-localization between the dyes, suggesting that the exosome preparation contains intact exosomes surrounded by membranes (Fig 9Ac). Next, these exosomes were incubated with *T*. *brucei* wild-type cells, and after incubation for 10 min, were visualized by ImageStream. The results (Fig 9Ba) indicate that exosomes (yellow) were found inside the cells. The analysis by ImageStream instrument also indicated a high level of internalization (Fig 9Bb). Green puncta which may represent exosomes within the endocytic pathway were also observed. It was recently suggested in mammalian cells that when exosomes are incubated with recipient cells, they do not accumulate at the cell surface, but enter the cells within minutes and are transported as intact vesicles within endosomes. The majority of these internalized exosomes move towards lysosomal compartments [57]. To verify that exosomes containing ZC3H41-GFP enter the endocytic pathway, cells were incubated with exosomes and stained with lysotracker. The results (Fig 9C) demonstrate clear localization of exosomes near or inside lysosomes and establish that exosomes secreted from *SmD1* silenced cells can enter recipient trypanosome cells.

### Exosome secretion enhances the repulsion between parasites engaged in social motility

Since exosomes were shown to participate in cell to cell communication in cancer, we explored if similar effects exist in *T*. *brucei*. The only cell to cell communication mechanism known thus far in trypanosomes is social motility (SoMo) [24, 58–60]. The recently reported social motility of trypanosomes in culture may mimic the migration of procyclic parasites in the insect gut [24, 25]. Indeed, *T*. *brucei* cells placed on semi-solid media were shown to migrate, and form radial projections from a central colony, a structure implicated in social motility [24, 25]. However, different migrating populations never mix, and the parasites remain as separate communities [24, 25].

**Fig 10:**
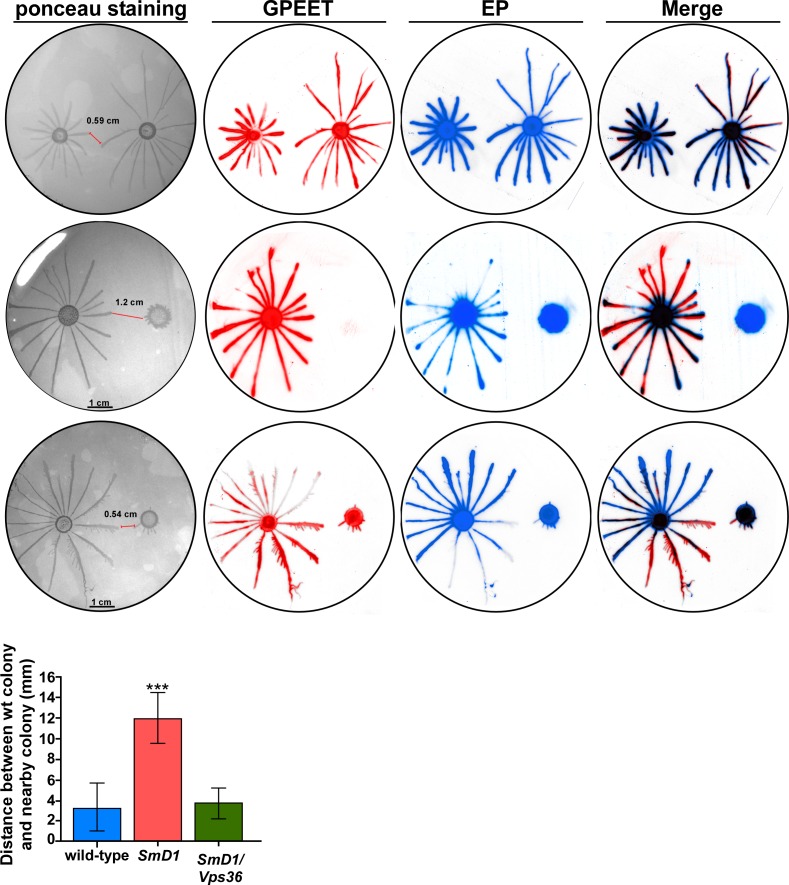
Exosome secretion repels the migration of wild-type cells. **Cells (~10**^**7**^**) were plated on semi solid agar containing tetracycline at a distance of 2.5 cm from each other.** The *SmD1 and SmD1/Vps36* silenced cells were plated on plates containing tetracycline. Pattern formation was analyzed 2 days after plating. The parasites from the plates were blotted, and the blot was stained with Ponceau and then reacted with anti-GPEET and anti-EP. The distance between the wild-type colony and the adjacent colony is presented. The statistical analysis represents the mean ± s.e.m **P< 0.01 comparing the distance between wild-type to wild-type, wild-type to *SmD1and* wild-type to *SmD1/Vps36* silenced cells based on three independent experiments.

To examine if exosome secretion might effect SoMo, the movement of parasite colony was examined on semi-solid agar. Cells were plated at equal distance (2.5 cm) from each other. It was recently shown that only early PCF cells, which express the GPEET surface protein are able to engage in social motility, while late PCF cells, expressing only EP procyclin lose this capability [25]. Migration of cells was followed macroscopically (Fig 10). The cells were then transferred to nitrocellulose, stained with Ponceau, and reacted with antibodies to GPEET and EP. When two wild-type populations were spotted, they migrated towards each other, but stopped their migration at distance of ~0.59 cm. *SmD1* silenced cells do not express GPEET, and hence were unable to engage in social motility as expected. Interestingly, these cells secreting exosomes are sensed by the wild-type cells, and wild-type cells stop their migration towards these cells at distance ~1 cm. If exosomes are indeed the entities that transmit the information to the wild-type migrating cells, then *SmD1/Vps36* cells should not be able to send such inhibitory signals. *SmD1/Vps36* silenced cells do express GPEET, possibly because the double-silencing is not as efficient as silencing of cells expressing only the *SmD1*-silencing construct (explained above). However, these cells do not secrete exosomes. Indeed, wild-type cells approached the *SmD1/Vps36* silenced cells as closely as they approached the wild-type cells, suggesting that exosome secretion is involved in the repulsion mechanism between these two parasite communities. Note that the distance was always measured from the tip of the wild-type projection to the tip of the nearest silenced cells (indicated in the figure with a red line).

Exosomes are not the only factor(s) controlling this repulsion; cells that do not secrete exosomes, such as the *SmD1/Vps36* silenced cells, maintained a distance similar to that observed between two wild-type populations (~0.5 cm), suggesting that additional factor(s) secreted from cells help the two populations avoid mixing. The experiments in (Fig 10) were repeated three times and the difference in the distance between (*SmD1* or *SmD1/Vps36*) and wild-type cells was statistically significant (p< 0.01**).

**Fig 11:**
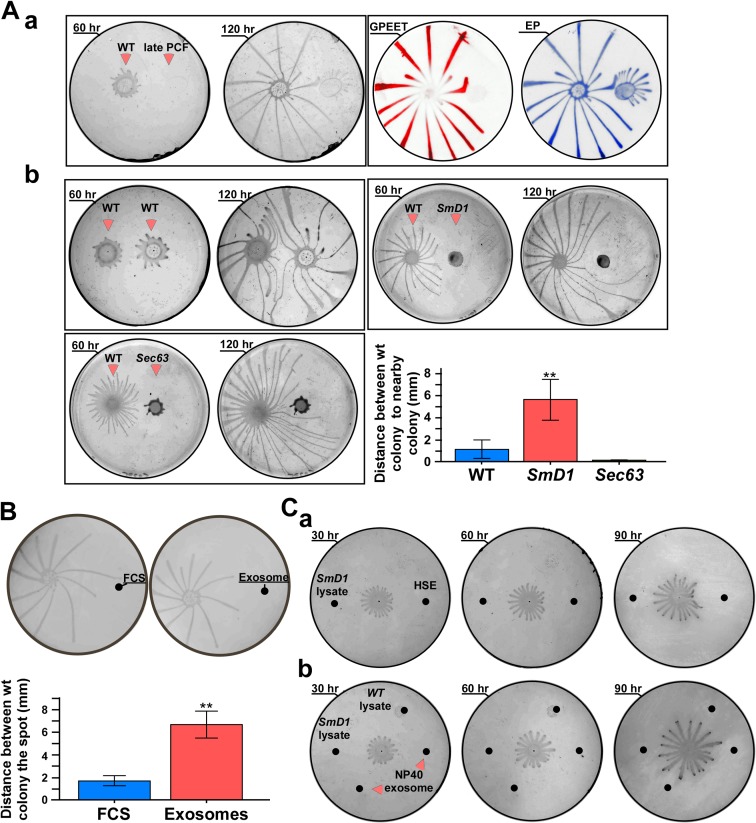
**(A) Effect on wild-type migration by different cell type. (a) Late PCF cells do not express GPEET.** Cells were plated on semi solid agar at a distance of 2.5 cm from each other. The plates were subjected to western analysis with GPEET and EP antibodies. The identity of the cells and incubation time are indicated. **(b) Migration of cells following longer incubation on plates**. Cells were plated on semi solid agar containing tetracycline at a distance of 2.5 cm from each other. For longer time points, 8×10^5^ cells (in 5 μl) were used. Wild-type, *SmD1* and *SEC63* were silenced on plates for the time indicated. Statistical analysis represents the mean ± S.E.M **P< 0.01 comparing the distance between wild-type cells and the silenced cells (based on three biological replicates). **(B) Free exosomes but not FCS divert the migration of parasites.** Cells (~10^7^) were plated on semi solid agar containing tetracycline at a distance of 2.5 cm from each other. The plates were seeded with either a solution of FCS (5 μg/μl) or with semi-purified exosomes (5 μg/μl). The drops (5 μl) of FCS and of exosomes (5 μl of extract from 10^9^ in 200μl) were added at the same spot every 12 hrs. for 3 days; scale bar, 5 mm. Statistical analysis represents the mean ± S.E.M **P< 0.01 comparing the distance between wild-type cells and exosomes versus wild-type cells and FCS (based on three biological replicates). **(C) Damaged exosomes or protein lysates do not affect the migration of cells. (a)** Migration of wild-type cells was monitored on plates that were seeded with exosomes prepared from cells subjected to heat-shock (HSE) (5 μl, exosomes prepared from 10^9^ cells i.e. 25 μg), and 25 μg of lysate prepared from *SmD1* silenced cells; **(b)** the same as in **(a),** but the plate was seeded with 25 μg of lysate prepared from *SmD1* silenced cells, lysate from wild-type cells, and exosomes which were pre-treated with NP40. The exosomes (1/10 purified from 10^9^ cells) from *SmD1* silenced cells (25 μg) were mixed with NP40 to 0.05% and were incubated over night before seeding on the plates. The plates were incubated for the indicated times. The position to which the substances were seeded are marked with black spots. The experiment was repeated three times.

However, since *SmD1* silenced cells are late PCF and *SmD1/Vps36* also contain early procylic cells, it was important to examine if the effect observed is not due to this difference. To this end, we compared the migration of wild-type cells towards cells which are "locked at" the late PCF state (kindly provided by Isabel Roditi, Bern Switzerland). Indeed, these cells do not engage in social motility, are GPEET negative, and EP positive (Fig 11Aa) and form only very small projections after 5 days on plates. When comparing the repulsion of wild-type cells to *SmD1* silenced cells versus that of only late PCF cells, we found that *SmD1* silenced cells (Fig 11Ab) are more strongly repulsive than late PCF cells (Fig 11Aa). Thus the repelling activity of *SmD1* silenced cells is not related to their late PCF state.

Next, we ruled out the possibility that the repelling activity may be due to the fact that *SmD1* silencing has a very severe growth phenotype, and compared the *SmD1* to *SEC63* silenced cells. *SEC63* silenced cells induce SLS. In this process, the SL RNA transcription is shut-off and the cells die by programmed cell death [53, 61]. The *SEC63* silenced cells secrete exosomes but in very minute amounts compared to *SmD1* silenced cells (S7 Fig). The distance between *SEC63* silenced cells and wild-type cells was shorter than that between wild type and *SmD1* silenced cells (Fig 11Ab). Finally, to ensure that the difference between *SEC63* and *SmD1* silenced cells in repelling the wild-type cells does not stem from changes in the viability of cells in the colony, cell viability was examined by live imaging (S1 Video, S2 Video). The results demonstrate movement of the silenced cells on the plate even ~100 hrs. post-plating. Thus, the repelling activity of *SmD1* silenced cells is not due to the death of these cells as a result of silencing.

In this experiment the cell trajectories were recorded (Fig 11Ab) for longer time points compared to Fig 10. The results (Fig 11Ab) indicate major difference between the migration of wild-type towards wild-type cells, as compared to migration of wild-type cells towards *SmD1* and *SEC63* silenced cells over time. The most striking result is the finding that wild-type cells divert their mobility and eventually bypass the *SmD1* silenced cell, but these cells always maintained longer distances from the colony compared to the distance to wild-type and *SEC63* silenced populations. The *SmD1* silenced cells generate a zone to which migrating cells do not enter. Interestingly, wild-type cells bypass the *SEC63* silenced cells, but the projections remain much closer to the colony. Thus, the data further support our contention that exosomes secreted from *SmD1* silenced cells play a role in transmitting repulsive signals.

Next, we examined whether these signals are cell autonomous, and if semi-purified exosomes act in a manner similar to cells expressing exosomes. Exosomes purified from *SmD1* silenced cells were placed 3 cm away from the migrating wild-type cells. Fetal calf serum (FCS) (containing the same amount of protein) was introduced at the same distance and was used as a control. Pure exosomes diverted the migration of the parasites, while FCS had no effect. Thus, the repulsive activity is not due to a physical barrier such as protein, since FCS did not have an inhibitory effect on SoMo (Fig 11B). A statistical analysis of three independent experiments is presented.

To examine if the biological activity of the exosomes requires that the exosome be intact, or whether their released contents can also be active in repulsion, extracts derived from *SmD1* silenced cells were placed at the same distance as exosomes prepared from cells which were exposed to heat-shock. Growth was monitored on plates, and the results (Fig 11C) clearly indicate that intact exosomes but not cell lysate divert the motility of wild-type cells. Similarly, exosomes that were disrupted with NP40, or lysate from *SmD1* silenced cells did not divert the motility of wild-type cells. The disruption of exosomes with the NP40 treatment was confirmed by analysis in NanoSight showing that the treatment eliminated the 100 nm exosome peak (S10 Fig). Thus, no biological activity was found following any of these treatments, and the disrupted exosomes lost their biological activity.

### Time-lapse microscopy demonstrates that exosomes deliver repulsion signals

**Fig 12:**
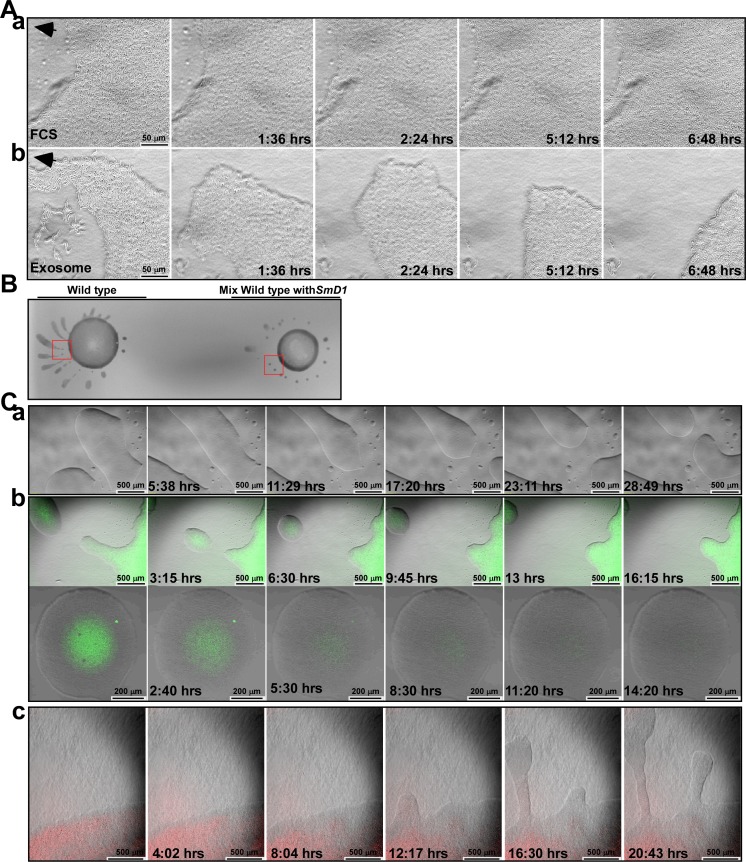
Wild-type cells crawl over FCS but “escape” from exosomes. **(A) Wild-type cells were plated on semi-solid agar and incubated 3 days while projections fully developed.** The cells at the edge of the projections were analyzed by time-lapse microscopy. **(a)** FCS (10μg) was placed at the edge of one projection (S3 Video); **(b)** exosomes (10 μg) were placed at another projection, and cell movement was monitored (S4 Video). The images taken from S3 Video **(a)** and S4 Video **(b)** are presented at the indicated time points following spotting of FCS or exosomes. Scale bar and time points of recording are indicated. **(B) Colony morphology of wild-type cells and a mixture of wild-type and *SmD1-*silenced cells.** Wild-type cells or a mixture of wild-type and *SmD1* silenced cells (silenced for 2 days prior to plating) were plated, and the morphology of the colony was recorded after plating. (**C) Time lapse microscopy: (a) The social motility of wild-type cells.** Wild-type cells were monitored) S10 Video. The images taken from this video are presented at the indicated time points. **(b) Effect of cells secreting exosomes on the social motility of wild-type cells.** Wild-type and *SmD1* silenced cells (silenced for 2 days prior to plating) expressing ZC3H41- GFP (green) were plated as described in Materials and Methods. S11 Video illustrates the effect of *SmD1-*silenced cells on the development of projections. The upper panel shows images that were taken from this video at the indicated time points. Scale bar and time points of recording are indicated. The lower panel (4X magnification) is at higher magnification showing the “balls” formed by these cultures. Images derived from (S12 Video) at the indicated time point are presented. Scale bar and time points of recording are indicated. **(c) Wild-type cells were mixed with *SmD1/Vps36* silenced cells (2 days prior to plating) expressing HIS2-RFP.** The cells were monitored for 20 hr (S13 Video). The images taken from this video are presented at the indicated time points. Scale bar and time points of recording are indicated.

To further understand the effect on migration, we sought to monitor the repulsion of social motility in real-time by time-lapse microscopy. Parasites were followed microscopically for 72–80 hrs. post plating. FCS was placed at the edge of the projection and motility was observed for 8 hrs. The migrating crowd ignored this protein deposit, and cells covered the area where the FCS was originally placed (Fig 12Aa S3 Video). In contrast, when exosomes (containing the same amount of protein) were placed at a different projection of the same colony and visualized for the same amount of time, a different phenotype was observed. The cells first moved forward but then retracted back (Fig 12Ab, S4 Video). Thus, our results further suggest that the exosomes secreted from the *SmD1* silenced cells transmit a repulsive signal.

Likewise, NP40 damaged or heat-damaged exosomes, were placed near the tip of the projection and their effect on the social motility was examined in comparison to migration within an untreated projection. The results presented in (S5, S6 and S7 Videos) clearly indicate that cells did not escape from any of these treatments, but escaped from exosomes secreted from cells under heat-shock (S8 Video) suggesting that damaged exosomes lost their biological activity. In addition, no repulsive activity was observed with lysates made from *SmD1* silenced cells (S9 Video). These results suggest that only intact exosomes are able to transmit signals to the migrating cell population.

Finally, we examined how wild-type cells would react when mixed with cells secreting exosomes. Wild-type cells form projections by merging of cell clusters. When wild-type cells were mixed with *SmD1* silenced cells at a 1:1 ratio, the mixed population failed to form radial projections, but formed ball-like structures, which never merged (Fig 12B).

It was previously reported that parasites migrating on an agar plate move *en masse*, recruiting cells, and enabling the merging of groups of cells into ball-like structures. These balls merge to form the projections radiating from the center of the colony (Fig 12Ca, S10 Video). When the behavior of a mixed culture of wild-type and *SmD1* silenced cells was followed by time-lapse microscopy, a completely different pattern was observed. The *SmD1* silenced cells expressed ZC3H41-GFP and could be distinguished from the wild-type cells. The results presented in Fig 12Cb and S11 Video, demonstrate that plating of the *SmD1* silenced cells with wild-type cells affected the social motility of the wild-type cells and prevented the formation of projections. Balls of cells containing the silenced cells in the middle and the wild-type at the periphery were released from the dense population at the site of inoculation, but these balls never merged to form projections (Fig 12Cb, S11 Video). Eventually, the ZC3H41-GFP *SmD1* silenced cells disappeared either due to natural death or maybe because wild-type cells produced toxic substances to kill these cells (Fig 12Cb, S12 Video). However, despite the disappearance of these *SmD1* silenced cells the wild-type cells never formed normal projections. This phenotype is caused by exosome secretion, since *SmD1/Vps36* silenced cells tagged with HIS2-RFP did not affect the migration of the wild-type cells, despite being moribund. These red cells were left behind, but their presence did not affect the migration of wild-type cells (Fig 12Cc, S13 Video). These data further support our findings that the repulsive signals sent to the migrating population are transmitted via exosomes.

## Discussion

In this study, we describe a novel process demonstrating that exosomes secreted from trypanosomes that are under stress due to inhibition of *trans-*splicing affect social motility. Migrating parasites avoid contact with these exosomes, and wild-type cells are repelled from exosome-secreting cells. Moreover, while the presence of cells secreting exosomes distorted the normal migration of wild-type cells, when exosome secretion was inhibited, no effect on the migration of wild-type cells was observed, strongly suggesting that exosomes are responsible for sending the repellent signal(s). Only intact exosomes possess this biological activity. Exosome secretion is mediated by the ESCRT machinery, and their secretion pathway is distinct from the secretion of EVs recently described in trypanosomes [23]. The markers that classify these exosomes are SL RNA and the helicase ZC3H41.

### Why is SL RNA secreted via exosomes?

It was not immediately apparent why trypanosomes developed a dedicated mechanism to stabilize SL RNA only to subsequently secrete it via exosomes. In this study we investigated the intriguing possibility that exosome secreting SL RNA, as well and possibly additional proteins and RNAs are used by the parasites to transmit signals to neighboring parasites. Interestingly, the proteins which we showed to stabilize the cytoplasmic SL RNA are not related to known RNA processing or RNA degradation factors. These factors might be related to the stress response induced by extinguishing *trans*-splicing. However, exosome secretion under these conditions might be an example of a more general phenomenon whereby exosome secretion is used by cells to deliver repulsive signals especially when a basic and essential process like *trans*-splicing is compromised. However, the SL RNA might not itself be the repellent, but rather a marker for these exosomes.

### Extracellular vesicles in trypanosomes

This study provides the first functional evidence that exosomes in *T*. *brucei* are formed in MVBs using the ESCRT machinery. These vesicles were found inside MVB structures, and silencing of *Vps36* compromised the secretion of SL RNA via exosomes.

The mode of secretion of the exosomes observed in this study differs from the mechanism suggested for formation of the nanotube-derived EVs, which bud from the flagellar membrane. Although exosomes were found around the flagellar pocket, this is most probably not the only exit site for secretion, as the exosomes are evenly distributed on the surface of the parasite. The multiple secretion routes are in contrast to exocytosis and endocytosis, which are believed to take place in trypanosomes only from the flagellar pocket [62]. Indeed, in *T*. *cruzi*, different types of secreted vesicles were reported, as well; based on their protein composition, it was suggested that the different vesicles originate from plasma membrane, MVB, and even through autophagy-based secretion [47]. Secretion from the surface of the cell could also be mediated by ectosomes. Ectosomes are extracellular vesicles that are generated by outwards budding from the plasma membrane followed by pinching off and release to the extracellular space. Ectosomes release also requires the ESCRT machinery, and especially VPS4 [63]. It remains possible that the massive secretion from the entire surface of the parasite seen in this study also occurs through ectosome secretion.

Although exosomes were initially viewed as a mechanism to discard unwanted proteins [14, 64], this concept was changed with the first exciting finding describing the role of exosomes in secreting microRNAs [12]. Exosomes were also shown to help pathogens evade the immune system [16, 17, 21]. Studies in malaria demonstrate that exosome-like vesicles are involved in cellular communication, sense population density, and regulate the balance between asexual growth and production of gametocytes [16, 19, 20]. Interestingly, the formation and biogenesis of secreted malarial vesicles is different from that of the exosomes described in this study, since malaria parasites lack ESCRT I and II components [16, 51]. *Leishmania* also uses exosomes to deliver the GP63 protease to hepatic cells, where they inhibit Dicer1, blocking host microRNA processing, which affects parasite burden [18]. High temperature was shown to induce exosome secretion from *Leishmania* [45, 46], as we report here. Most recently, it was demonstrated that *Leishmania* secrete exosomes within the lumen of the sand-fly midgut, and that exosomes co-injected with *Leishmania* exacerbate lesions in a footpad model due to the over induction of inflammatory cytokines mediated by the exosomes [44].

Recently, the shedding of EVs, which can fuse both with neighboring parasites and host cells, was described in *T*. *brucei* [23]. It was suggested that the EVs transmit signaling proteins both to parasites and to myeloid cells, which govern innate immunity. These EVs also fuse with erythrocytes and enhance their clearance, inducing anemia [23]. Thus, EVs can modulate both innate immunity and control pathogenicity within the host. EVs were described in the bloodstream form [23]. This study demonstrates their presence in PCF parasites, as well. It is possible that the EVs secreted by procyclic parasites modulate the immune response of the tsetse fly vector. Indeed, evidence exists for the involvement of antimicrobial peptides in trypanosome transmission in tsetse [65]. In addition, the TsetseEP protein, an immune-response protein, which is present in the midgut, hemolymph and salivary glands, was shown to be involved in innate immunity [66]. However, the exosomes described in this study seem to be different from these EVs, since exosome secretion is blocked by silencing of *Vps36*, while the nanotubes are still formed and released. Future studies should identify the secretion mechanism of these nanotubes. Only then, will it be possible to assess their role in affecting the innate immunity of the host. The data presented here demonstrate that exosomes containing SL RNA (but not only) are distinct from EVs and stress granules. In addition, SL RNA-containing exosomes are the most abundant ones secreted when *trans*-splicing is inhibited. Thus the biological phenomenon observed in this study could be attributed to these SL RNA-containing exosomes. In support of the role of SL RNA, the exosomes secreted from *SEC63* silenced cells did not have the same repelling biological activity as those secreted from *SmD1* silenced cells. Note, that the proteome of *SEC63* silenced cells shares changes which are common to *SmD1* silenced cells, possibly because these two type of cells are inhibited in *trans*-splicing. However, changes that are specific to each of these perturbations were also identified [54]. The repelling activity of these two silenced cells clearly indicate that the repelling signal(s) is not specific to dying cells (Fig 11). The wild-type cells specifically maintain a “zone of no entry” only from the *SmD1* but not from the *SEC63* silenced cells. Only *SmD1* silenced cells which are actively secreting exosomes continuously repel a migrating population. The repelling activity revealed in this study cannot be attributed solely to the fact that these cells are late procylics, because unsilenced late PCF cells are not as efficient as *SmD1* silenced cells in repelling a migrating population (Fig 11). We demonstrated here that exosomes secreted from cells enter recipient trypanosome cells. Future studies will involve the selection of these cells and analysis of changes in their transcriptome and proteome, which are likely to reflect their social behavior. Such cells may be selected in future studies using the tools generated here, either by enriching these recipient cells or by performing single-cell analysis.

### Social motility in trypanosomes and its possible regulation by exosomes

Social motility was described in procyclic parasites, although its biological role is still unclear [3]. Similar SoMo phenomena were observed in certain bacteria [67]. Indeed, *T*. *brucei* social motility [24] could coordinate the movement of the parasites during their migration within the fly, and it was recently demonstrated that SoMo is a property typical of early procyclic parasites, which express the surface protein, GPEET. SoMo is therefore an early event in the colonization of the insect host, most likely occurring during the migration from the endotrophic space to the ectoperitrophic space within the midgut [25]. We propose that exosome secretion under stress (e.g. inhibition of *trans-*splicing or heat-shock) is part of the repulsive mechanism that is used to ensure the well-being of parasite communities or individuals. More studies are needed to determine if parasites move individually to the ectoperitrophic space or as a cohort governed by social motility cues, similar to that observed on plates.

On plates, individual *T*. *brucei* cells first collect into colonies at the inoculation sites; the colonies then move as groups, and these groups are able to collect individuals from their surroundings [24]. Cells secreting exosomes emit repulsive signals that preclude their joining a migrating population, thereby preventing unfit individuals from engaging in social motility. This concept was clearly demonstrated also here using time-lapse microscopy. Only cells secreting exosomes, but not cells which are unable to secrete exosomes affect the social motility of wild-type cells. We suggest that only intact exosomes transmit repulsive signals. Although the repulsive signals might exist in cell lysates prepared from *SmD1* silenced cells, these cannot execute their biological activities because the intact exosomes appear to be required as a vehicle for their delivery (especially RNA molecules) (Fig 11). Although the hallmark of these exosomes is the presence of SL RNA, we have no evidence thus far to prove that it is the SL RNA per-se which serves as the repellent. It is possible that the signal is complex and involves SL RNA and additional RNA or protein factors. The cap-4 modification does not play a role in this signaling, since SL RNA lacking the +4 cap modification which is secreted via exosomes from *SmD1* silenced cells, or fully modified SL RNA, secreted via exosomes under heat-shock, are both found in active exosomes. Interestingly, repulsive factors are secreted from cells in which the *trans*-splicing process is perturbed, while these cells lost their ability to synthesize or perceive the positive migrating signals.

A recent study suggested that EVs may function to help *T*. *brucei* regulate infection within the host [23]. Indeed, EVs were shown to contain adenylate cyclase, proteases, and other known virulence factors, which support the survival of parasites within the host [23]. For instance, GRESAG4 was found in EVs [23]; this protein was previously proposed to increase the cAMP levels in host immune cells, leading to reduced production of TNF-alpha, and thus protecting the trypanosome from the immune response [68]. However, cAMP was also shown to affect social motility [30, 31]. Thus, these two types of *T*. *brucei* vesicles, EVs and exosomes, which exist on the cell surface, may serve different biological functions. This study further demonstrates that these EVs and exosomes are biochemically different.

Very relevant to this study is the recent study demonstrating that *Leishmania* secretes exosomes within the lumen of the sand fly midgut [44]. In fact, the exosomes are part of sand fly inoculum and are co-egested with the parasite during the insect’s bite, affecting host infection. Indeed, exosomes were able to exacerbate lesions mainly though over induction of inflammatory cytokines. Thus *Leishmania* exosomes should be included into the repertoire of virulence factors [44].

Parasites must scan their environment and activate signaling pathways that will correctly balance propagation and transmission in the hosts. This study highlights the role of exosome secretion as a marker of the well-being of these parasites. Under certain stresses, parasites use exosome secretion to send indications of their unfitness or about their immediate stressful environment. We do not yet know how the exosome signal is perceived in the recipient parasite cell. This should be the direction of future studies. Thus, exosomes, and EVs shed from nanotubes, assist in parasite communication with their hosts and among themselves, as demonstrated here. Perturbation of these communication routes could be harnessed as a novel strategy to inhibit parasite propagation and transmission.

## Materials and methods

### Cell lines and transformations

Lister 427 monomorphic *T*. *brucei*, procyclic form, were designated wild-type in this study. For silencing, the *T*. *brucei* 29–13 was used, which carries integrated genes for T7 polymerase and the tetracycline repressor. The silencing constructs using the T7 opposing and the stem-loop constructs were based on [36]. YFP and RFP-tagging was performed as in [69], and TAP-PTP as in [70].

### Extract preparation for affinity selection of cytoplasmic SL RNA

Cell extracts (150 mM) and post-ribosomal supernatant (PRS) were prepared, as described [9]. PRS were separated on a FPLC Superdex 200 gel filtration column, and fractions carrying SL RNA were subjected to affinity selection in the presence of 150 mM KCl, as previously described [33]. The proteins were directly analyzed on 12% polyacrylamide gels.

### UV cross-linking and affinity purification of SL RNA associated proteins

Cells (2×10^9^) were washed with PBS, re-suspended, placed 5 cm from a UV lamp, and irradiated at 254 nm for 5 minutes on ice. Cells were lysed as described above, and the extract was subjected to affinity selection in IgG beads, as described [10]. The bead pellet was treated with proteinase K in the presence of 1% SDS, and RNA, extracted and analyzed by primer extension.

### Primer extension and Northern analysis, *in situ* hybridization combined with immunofluorescence

Primer extension was performed as described previously [9]. *In situ* hybridization with SL RNA was performed as described [71]. SL RNA was detected with Alexa red streptavidin, and Alexa 488 conjugated-second antibody. The slides were incubated with 1:400 diluted primary ZC3H41 antibodies. Nuclei were stained using 4’-6’-diamidino-2-phenylindole (DAPI). Cells were visualized by a Nikon eclipse 90i microscope with Retiga 2000R (QIMAGING) camera.

### Generation of antibodies against ZC3H41

The full-length sequence of ZC3H41 was cloned into the pHIS vector, and antibodies were prepared in rabbits, as described [42].

### *In vivo* labeling

Procyclic cells were washed twice with PBS and resuspended to 10^8^cells/ml in pre-warmed (27°C) Methionine free RPMI (Sigma). The cells were labeled with (100 μCi) 1000Ci/mmole L-[^35^S]Methionine (IZOTOP, Hungary) for 10 minutes followed by a 5 min chase.

### Exosome purification

Parasites were grown in FCS depleted of exosomes. To remove exosomes FCS was subjected to two centrifugations at 10, 000 ×g for 15 hrs. Exosomes were prepared from S*mD1*-silenced (10^9^ cells), grown in exosome-free FCSafter 48hrs. of silencing. After removing the cells by centrifugation the supernatant was filtered through a 0.45 μm filter and the filtrate was subjected to ultracentrifugation at 100,000 ×g at for 24hr in a T1270 rotor. The pellet was washed with PBS and subjected to a second ultracentrifugation as described above. For separating exosomes on sucrose gradients, the pellet (crude exosomes) was resuspended in 250 μl of PBS (~1.5 mg protein), loaded on a 15–50% sucrose gradient and centrifuged at 39,900 rpm (210, 000 ×g) for 20 hrs. in an SW41 rotor. Fractions (500 μl) were collected and their refractive index was determined. Fractions were visualized by SEM or subjected to western analysis and primer extension.

### ImageStream analysis

Cellular fluorescence was captured and photographed using an ImageStreamX high-resolution imaging flow cytometer (Amnis, Co., Seattle, WA). Approximately 1000 cell samples were excited using two lasers; 561 nm for DiL (Thremo Fisher, Waltham, MA) and 488 nm (for GFP). Following the flow cytometry image capture, samples were gated to attain populations of captured single-cell images of living cells that were in focus. The image pairs obtained from the two emission modes were used to quantify the co-localization of two probes, in a defined region. A co-localization parameter can thus be used to assess whether the two different signals are located in the same area or very near to one another.

### NanoSight analysis

Exosomes preparation were analyzed in NanoSight NS300 instrument (NanoSight Ltd. Amesbury, UK) after 1:1000 dilution of the sample yielding particle concentration in the range of 1×10^6^ particles/ml.

### Transmission electron microscopy (TEM)

Cells (2×10^7^) were fixed in Karnovsky fixation solution (0.5% glutaraldehyde and 2% formaldehyde). Ultra-thin sections were cut, and the sections were stained with uranyl acetate and lead citrate. The preparations were examined using an FEI Tecnai 120Kv transmission electron microscope, as previously described.

### TEM of exosomes

Isolated exosomes prepared as described above (2.5 μl derived from ~10^9^ cells) were dried onto freshly glow discharged 300 mesh formvar carbon-coated TEM grids, negatively stained with 2% aqueous uranyl acetate and observed with a FEI Tecnai G2 120Kv transmission electron microscope.

### Scanning electron microscopy (SEM)

Cells (2×10^5^) were fixed in Karnovsky solution, and slides were incubated with tannic acid and then with 4% OsO_4._ The samples were dehydrated using Freon. The samples were coated with carbon, and visualized using an FEI Quanta 200 FEG at 20 kV electron microscope.

### Immonugold SEM

Cells (2×10^5^) were placed on 10 mm cover glass and fixed with 4% formaldehyde for 20 min. The slides were blocked in 10% FCS, and then incubated with anti-ZC3H41 (1:400). After washing, the slides were incubated with anti-Rabbit gold conjugates (size ~18nm). After extensive fixation the SEM protocol was performed but omitting the fixation with OsO_4_. The samples were visualized by FEI Quanta 200 FEG at 20 kV with backscattered electron detector.

### Immunogold labeling and electron microscopy analysis by TEM

Cells were fixed in 4% paraformaldehyde with 0.1% glutaraldehyde in 0.1M cacodylate buffer (pH 7.4). The samples were soaked in 2.3M sucrose solution, and rapidly frozen in liquid nitrogen. Frozen ultrathin (70–90 nm) sections were cut with a diamond knife at −120°C on a Leica EM UC6 ultramicrotome. The sections were collected on 200-mesh Formvar coated nickel grids. Sections were blocked, and immuno-labeling was performed using ZC3H41 antibodies (1:50), or with GFP antibodies which cross-react with YFP. (1:30; GFP antibodies (FL) from Santa Cruz Biotechnology), followed by incubation with goat anti-Rabbit IgG coupled to 10-nm gold particles (1∶20, Jackson Immunoresearch, USA). Contrast staining and embedding were performed as previously described [72]. The embedded sections were viewed and photographed with an FEI Tecnai SPIRIT (FEI, Eidhoven, Netherlands) transmission electron microscope operated at 120 kV, and equipped with an EAGLE CCD Camera.

### Motility assay and visualization

Cultivation on semi-solid agarose plates was performed using a protocol adapted from [73]. Plates were prepared as described in Imhof et al., 2014 [25]. To inoculate the parasites, 5 μl of cell suspension (1.5×10^7^cells/ml) was spotted onto the agarose surface, and the plates were sealed with parafilm and incubated at 27°C. Colony lift was performed as described in [25]. The blots were stained with Ponceau and then reacted with antibodies to EP [Cedarlane (USA) 1:1000)] or GPEET (1:1000). Antibody binding was detected with goat-anti-rabbit IgG or anti-mouse IgG coupled to horseradish peroxidase, and visualized by ECL.

### Time lapse microscopy

The plate was imaged using an Olympus UCBIX81 microscope with an UPLAN objective and XM10 CCD camera at room temperature. Images were captured at using Olympus cellSens dimension software. The videos were recorded in real time in VSI format and digitalized in AVI using Image J 1.46r.

Culture fluid (5 μl) containing 2×10^8^ cells of each type were plated on the solid medium and microscopic inspection started 30 minutes after plating. The forward movement of cells was observed as indicated in the different experiments. Imaging was performed using an Olympus UCB / IX81 microscope in with an UPLAN objective and XM10 CCD camera.

## Supporting information

S1 Fig Purification of cytoplasmic SL RNA complex. Purification was performed as described in Fig 1A. Fractions containing SL RNP-C obtained from 5×10^9^ cells and fractionated on Superdex S-200 column were affinity selected as described in Materials and Methods. The proteins were extracted from the streptavidin beads, separated on a 12% acrylamide SDS gel, and stained with silver. The designation of the proteins is indicated. Panels a-d represent four independent purifications. The most prominent bands that were further studied and do not appear in the (-Oligo) preparation are indicated.
(PDF)

S2 Fig Sequence alignments were performed using ClustalW multiple sequence alignment program (http://www.genome.jp/tools/clustalw/). The alignment with the trypanosomatid species; *Trypanosoma brucei* (Tb), *Trypanosoma cruzi (*Tc), and *Leishmanina major* (Lm), are presented. Residues shown in red, blue and green represent identity, similarity and weak similarity, respectively. The sequences were obtained from GeneDB. **(A)** Sequence alignment of *T*. *brucei* p22 (Tb927.7.7460) with its homologues. The PDIb (protein disulfide isomerase) is indicated. **(B)** Sequence alignment of *T*. *brucei* p72 ATPase (Tb927.3.1590) with its homologues. **(C)** Sequence alignment of *T*. *brucei* ZC3H41 (Tb927.11.1980) with its homologues. The different domains are indicated; SAM, sterile alpha motif; ZF, zinc finger; KH, K homology domain.
(PDF)

S3 Fig Co-silencing of *SmE* with either *p22* or *p72*. **(A)** Western analysis. Cells expressing the T7 opposing silencing constructs for *SmE/p22* and tagged PTP-p22 construct, or *SmE/p72* with the PTP-p72 tagged construct, were silenced for 48 hrs. Cells (~10^6^ cells/ lane) were subjected to western analysis using PTB1 antibodies, which also recognize the tagged protein. **(B)** The silencing of *p22* and *p72* affect SL RNP-C stability. Cells carrying the *SmE/p22* and *SmE/p72* silencing constructs were induced for 48 hrs. RNA (10 μg of total RNA) was subjected to primer extension with primers specific to SL RNA, U4, and U3 snoRNAs (listed in S2 Table). The extension products were separated on a 6% denaturing gel. The identity of the cell line and the position of the modified cap are indicated. The statistical analysis represents the mean ± s.e.m of quantification from three independent experiments. **P <0.01, and ***P <0.005 compared to–Tet, using Student's *t*-test. **(C)** Localization of p22 and p72 before and after silencing. Cells expressing PTP-p72 and the *SmD1* silencing construct, either un-induced or induced for the indicated times were fixed, and fluorescence was monitored. Nuclei were stained with DAPI.
(PDF)

S4 Fig Changes in localization of ZC3H41 and SL RNA during *SmD1* silencing. Cells carrying the *SmD1* silencing construct were induced for the times indicated and subjected to *in situ* hybridization with SL RNA (red), and IFA with ZC3H41 antibodies (green). The nucleus was stained with DAPI. The merge was performed on DAPI staining and SL RNA hybridization. The time points post-silencing are indicated.
(PDF)

S5 Fig MTR4 silencing **(A)** Northern blot analysis of cells carrying the silencing construct for *SmD1/Mtr4*. RNA was prepared from un-induced cells (-Tet) and cells after 2 days of induction (+Tet). Total RNA (20 μg) was subjected to Northern analysis with anti-sense RNA probe to *Mtr4* (Tb927.10.7440). The mRNA transcripts, dsRNA, as well as 7SL RNA are indicated. **(B)** Quantification of changes in SL and U3 snRNA. The ratio between SL RNA and U3 was calculated for each time point that is presented in Fig 2A (*SmD1* silenced cells) and in Fig 2B (*SmD1/Vps36* silenced cells). **(C)** As in **(B)** but showing the ratio between U2 and U3 snRNAs. **(D)** ZC3H41 is present mostly outside of P-bodies. ZC3H41 localization was determined with respect to P-bodies labeled with DHH1. Cells carrying the *SmD1* silencing construct and the YFP-DHH1 construct were silenced for 2 days and subjected to IFA using ZC3H41 and YFP antibodies (red and green, respectively). The nucleus was stained with DAPI. **(E)** Cytoplasmic SL RNA is not found in P-bodies. Cells carrying the *SmD1* silencing construct and expressing YFP-DHH1 were induced for 2 days and subjected to *in situ* hybridization with SL RNA (red), and immunofluorescence using YFP antibody for YFP-DHH1 (green). The nucleus was stained with DAPI. **(F)** SL RNA granules are distinct from stress granules. Cells were silenced for 2 days and stained by IFA using PTB1 antibodies (green stain) and subjected to *in situ* hybridization with SL RNA (red). The nucleus was stained with DAPI. **(G)** As in **F** but using antibodies to eIF4E-1. The merge was performed between DAPI staining, IFA and *in situ* hybridization.
(PDF)

S6 Fig TEM of *SEC63* silenced cells. Cells were fixed after 2 days of silencing, and ultra-thin sections were prepared. The different ultra-structures are indicated. M, mitochondrion; ER, enodoplasmic reticulum; A, double-membrane autophagosome; Scale bars are indicated.
(PDF)

S7 Fig Exosome detection by SEM of *SEC63* silenced cells. Cells carrying the *SEC63* construct were silenced for 2 days and then fixed and visualized under EM. The scale bar is indicated. Exosomes are marked with arrowheads.
(PDF)

S8 Fig Silencing of *Vps36* does not affect the accumulation of SL RNA; inhibition of growth induced by *SmD1* silencing. **(A)** Western analysis demonstrating the depletion of Vps36. Cells carrying the *SmD1* silencing construct and the PTP-Vps36 tagging, un-induced (-Tet) and 2 days after induction (+Tet) were subjected to western analysis. PTB1 was used to control for equal loading. **(B)** Northern analysis demonstrating the silencing of *Vps36*. RNA was prepared from un-induced cells (-Tet) and cells after 2 days of induction (+Tet). Total RNA (20 μg) was subjected to Northern analysis with anti-sense RNA probe to *Vps36*. The mRNA transcripts, dsRNA, as well as 7SL RNA are indicated. **(C)** SL RNA was elevated in cells silenced for *SmD1/Vps36*. Cells carrying the silencing constructs were induced for the times indicated. RNA was subjected to primer extension with SL RNA and U3 probes. The position of the cap nt is indicated. **(D)** Growth curves of *SmD1*, and *SmD1/Vps36* silenced cells. The identity of the cell lines and treatment are indicated.
(PDF)

S9 Fig Cells continue to grow normally after heat shock. Cells were subjected to heat shock (37°C for 40 min) and then returned to 26°C; growth was monitored in comparison to cells which were not subjected to heat-shock.
(PDF)

S10 Fig NanoSight analysis. Exosomes were prepared from *SmD1* silenced cells (10^9^) after 2 days of silencing. The exosomes were treated with 0.05% NP40 for one hour and then analyzed by NanoSight instrument. Untreated exosomes (red), and treated exosomes (blue).
(PDF)

S1 Table The oligonucleotides used are listed below. (PDF)

S2 Table The list of proteins identified by Mass-spectrometry. The list includes data from three independent purifications. The mass spectrometry data were analyzed using the Sequest 3.31 software (J. Eng and J.Yates, University of Washington and Finnigan, San Jose) searching against T. brucei TriTryp database version 5 (http://tritrypdb.org/tritrypdb/). To control for the selective affinity selection (-Oligo). The affinity selection protocol was performed using fractions containing the SL RNA complex purified on a FPLC column but in the absence of oligonucleotide. The proteins associated with the beads were separated on a gel for a short time capturing the entire protein population. The gel slice was analyzed by MS.
(PDF)

S1 Video Time lapse movie demonstrating the viability of the *SmD1* silenced colony 60 hrs. post-plating (http://www.biu.ac.il/LS/Articles/SocialMotility/Video S1.avi). (AVI)

S2 Video Time lapse movie demonstrating the viability of the *SEC63* silenced colony 60 hrs post-plating (http://www.biu.ac.il/LS/Articles/SocialMotility/Video S2.avi). (AVI)

S3 Video Time lapse movie demonstrating the migration of wild-type cells over seeded FCS at the edge of a colony projection (http://www.biu.ac.il/LS/Articles/SocialMotility/Video S3.avi). (AVI)

S4 Video Time lapse movie demonstrating the retraction of wild-type cells from exosomes at the edge of a colony projection (http://www.biu.ac.il/LS/Articles/SocialMotility/Video S4.avi). (AVI)

S5 Video Time lapse movie demonstrating the migration of the wild-type population on the plate (http://www.biu.ac.il/LS/Articles/SocialMotility/Video S5.avi). (AVI)

S6 Video Time lapse movie demonstrating the migration of wild-type cells over seeded damaged exosomes (marked with an arrow) which were exposed to high temperature (65°C for half an hour) (http://www.biu.ac.il/LS/Articles/SocialMotility/Video S6.avi). (AVI)

S7 Video Time lapse movie demonstrating the migration of wild-type over seeded damaged exosomes (marked with an arrow) which were treated with 0.05% NP40 (http://www.biu.ac.il/LS/Articles/SocialMotility/Video S7.avi). (AVI)

S8 Video Time lapse movie demonstrating the retraction of wild-type cells from exosomes isolated from cells under heat-shock (marked with an arrow) which were placed at the edge of a colony projection (http://www.biu.ac.il/LS/Articles/SocialMotility/Video S8.avi). (AVI)

S9 Video Time lapse movie demonstrating migration of wild-type cells over extract derived from *SmD1* silenced cells (25 μg) (marked with an arrow) which were placed at the edge of a colony projection (http://www.biu.ac.il/LS/Articles/SocialMotility/Video S9.avi). (AVI)

S10 Video Time lapse movie demonstrating migration of wild-type cells forming projections (http://www.biu.ac.il/LS/Articles/SocialMotility/Video S10.avi). (AVI)

S11 Video Time lapse movie demonstrating migration of wild-type mixed in 1:1 ratio with *SmD1* silencing cells expressing ZC3H41-GFP (http://www.biu.ac.il/LS/Articles/SocialMotility/Video S11.avi). (AVI)

S12 Video Time lapse movie demonstrating the “ball” formation in a mixed colony of wild-type and *SmD1* silenced cells expressing ZC3H41-GFP (http://www.biu.ac.il/LS/Articles/SocialMotility/Video S12.avi). (AVI)

S13 Video Time lapse movie demonstrating the migration of mixed population of composed of wild-type and *SmD1/Vps36* silenced cells expressing histone2-RFP (http://www.biu.ac.il/LS/Articles/SocialMotility/Video S13.avi). (AVI)
