# Supplementary material for: Exosome secretion affects social motility in Trypanosoma brucei
Source: PLoS Pathog. 2017 Mar 3;13(3):e1006245. doi: 10.1371/journal.ppat.1006245 (PMC5352147; doi:10.1371/journal.ppat.1006245)
Supplement: S1 Fig — Purification was performed as described in Fig 1A. Fractions containing SL RNP-C obtained from 5×10^9^ cells and fractionated on Superdex S-200 column were affinity selected as described in Materials and Methods. The proteins were extracted from the streptavidin beads, separated on a 12% acrylamide SDS gel, and stained with silver. The designation of the proteins is indicated. Panels a-d represent four independent purifications. The most prominent bands that were further studied and do not appear in the (-Oligo) preparation are indicated. (PDF) [file ppat.1006245.s001.pdf]

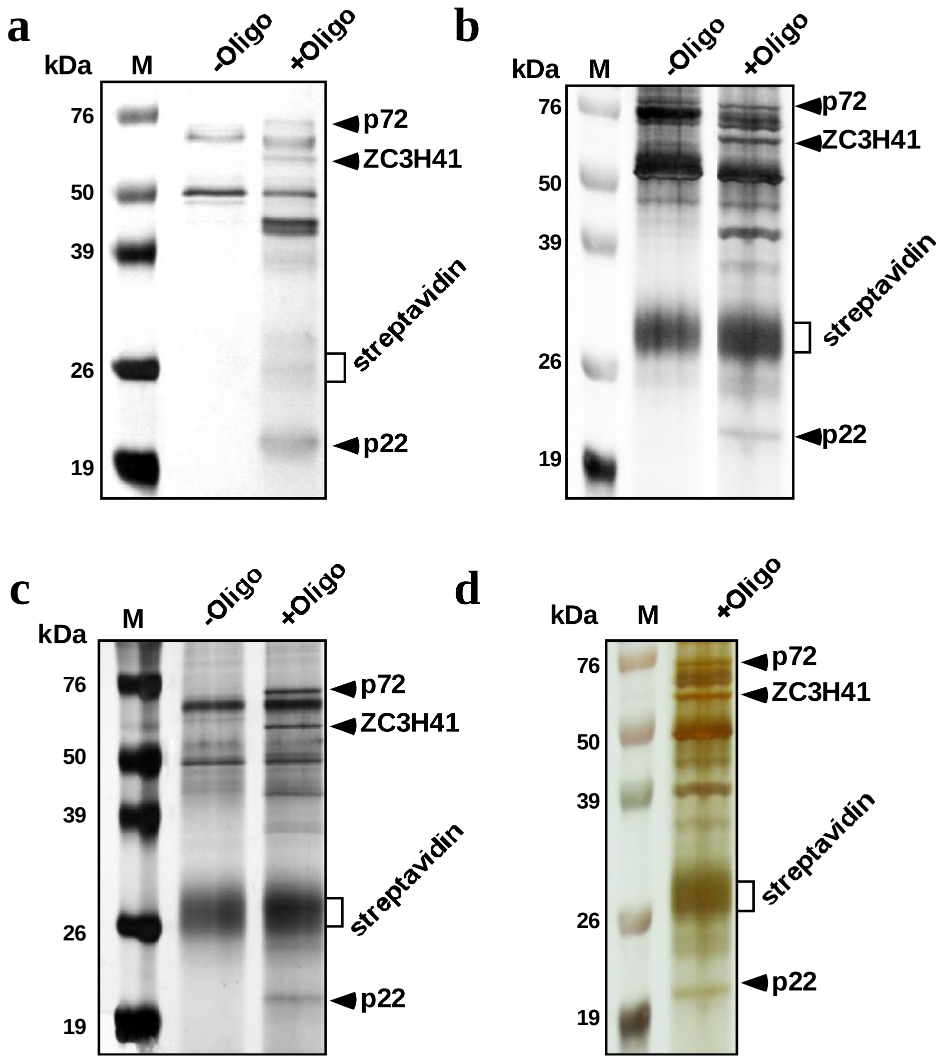

S1 Fig. Purification of cytoplasmic SL RNA complex. Purification was performed as described in Figure 1A. Fractions containing SL RNP-C obtained from 5×10^9 cells and fractionated on Superdex S-200 column were affinity selected as described in Materials and Methods. The proteins were extracted from the streptavidin beads, separated on a 12% acrylamide SDS gel, and stained with silver. The designation of the proteins is indicated. Panels a-d represent four independent purifications. The most prominent bands that were further studied and do not appear in the (-Oligo) preparation are indicated.
