# Supplementary material for: Exosome secretion affects social motility in Trypanosoma brucei
Source: PLoS Pathog. 2017 Mar 3;13(3):e1006245. doi: 10.1371/journal.ppat.1006245 (PMC5352147; doi:10.1371/journal.ppat.1006245)
Supplement: S2 Fig — The alignment with the trypanosomatid species; Trypanosoma brucei (Tb), Trypanosoma cruzi (Tc), and Leishmanina major (Lm), are presented. Residues shown in red, blue and green represent identity, similarity and weak similarity, respectively. The sequences were obtained from GeneDB. (A) Sequence alignment of T. brucei p22 (Tb927.7.7460) with its homologues. The PDIb (protein disulfide isomerase) is indicated. (B) Sequence alignment of T. brucei p72 ATPase (Tb927.3.1590) with its homologues. (C) Sequence alignment of T. brucei ZC3H41 (Tb927.11.1980) with its homologues. The different domains are indicated; SAM, sterile alpha motif; ZF, zinc finger; KH, K homology domain. (PDF) [file ppat.1006245.s002.pdf]

A

```
                                            PDIb
Tb    MVFS-THGIRVVYNTAN----VDRSLYAVVHCSDGKYAEVSKDHLNRLKFIEKSSEEEDI 55
Tc    MASF-TSCARILYNTTN----VDRTMHTIVHCSDGKYVEVPKAVADKCPFIEKVKEEMIP 55
Lm    MADEGAIDIRLVLKGPNGEFKVDRDLYIIVHCDDGKYIEVSKNYIKQCPFIEEA-EGEIP 59

Tb    EFDYPEAVLESLAQWTLKYGMDGVAVSSTVRPCIYRNVQHVLKDDWEREFFTTRLMNDLN 115
Tc    EFEYPAAVLENLVCWTEHYGIDGFAESEIVRPCIYRNILHVLKNKWDNGFFVSRLTSELN 115
Lm    EFGYPAAVLEHLIRWAVHYGVDGHAASQLTRPCIYRDFSYVVTDKWDNDFFNQRLCSPLN 119

Tb    IMHYLQTINAAEKYEMKGFHDFLCVCLSCKLRNEEDTELVHKIMGLEDFVEIGPSDLDEA 175
Tc    IGHYLQTVNAAEKFNMKGLHCFLCVGLSCKFRSEDDAELIHKIMGLPSNVQPNQEDIENV 175
Lm    QKHYLLTMTAAEQFGMQGLLDFMCIGLGCKLRGKDDNGIIHEVMGLDKEMEITSEDLAEV 179

Tb    ACRYTWLDQAIKPTVRR 192
Tc    TTRYPWLNEAIEPVVRK 192
Lm    SRDYPWFDDAVKATTKK 196
```

B

```
Tc    --MSYRAYG----GGYRGRGGRGGRGGQHWQQTACEEDTF-RDPSN-----FNSGSP--- 45
Tb    --MYHRGYG----GHYRGGRG-GGRGHSHWHNNAPMERGG-HMPSNNEGAAISSGSTG-- 50
Lm    MSSYYRGGSTRGRGHYRGGGEGGFRRGRGSYADAVSDGNMNRNEEQQRGERYRSQSSGDD 60

Tc    ----------------------YAELRNYFNSVEGVNYGELKSLTNASFALS-------E 76
Tb    ----------------------FSPLMDFFHSVEGRNYGELRSLTNETYQIS-------E 81
Lm    ESERGSGGFGGNPQCHAGPPLPLVALESFLRDVDGSNYSQLKQLTGRTYSLTRQMKPDPA 120

Tc    SVRCTFLAIQSDPFAPGSQVRMSCPCPFQLQKILQCSELSS---------------CVPC 121
Tb    NVRCTFLSIQSDPFAPGSQVRLVCPCTFSLEKVLQTTDLAA---------------ANPC 126
Lm    SVSIRFVRIQPDPFAPGSQVCVSVPAPFATHALLHSAHSTPPGVTAAALGNLLSPEDVAC 180

Tc    RRIAAEDYILRSFHDGHRRGVARHHSGALHVMRPSQHVLERSTVVLVNG---DRERGG-- 176
Tb    RRVAAEDFILRSFHAGYRNGIPRRTSGAVQVLRPSQHVLERSTVGLVKA---HQQKSGMQ 183
Lm    RRVAAEDFILRCVKGGFAAG--RNTSGAVQTIHQSQHVIARSAVRLLDASEAHDSDGTSD 238

Tc    VEVHLFARVKLPGHGRRIDGHGAIRIFYDELLPIMERCVVGLDEEALYQHVTCVHDQEHL 236
Tb    AEIEIFARVKLPGHGRRIDGHGAIDIFYNELVPLLEQCVVGLNEEDLHQHVICVHDQEVL 243
Lm    GWIHVYFRVKLPGHGRRIDGRRIQQILFSEVLPVFQENVLRCDHHALWAHVTCVHDQEWL 298
                                                        A-loop
Tc    RGELRAAGCVAFVANGSVLPRESGNSDRPLG-KGAVPFVSPASLERTFHLPHSGATVTGM 295
Tb    RSNLLGAGYVAFVANGAILPRDAGNSDKPLR-DNAVPFQSPKSLECSFTLPHSGKTITGM 302
Lm    RAQLREWGFAAFIADGAILPRATGNSDKPLSGPSVVAFSPPDTLRHTFQLPYSGRAISGA 358
                Walker A
Tc    GLPHGLILIAGGGFHGKSTLLRALEVGIYNHVPDDGRVYVTVDPTAVKIRAEDRRSINGV 355
Tb    GLPPGLTLIAGGGFHGKSTLLRALEVGIYNHVPDDGRTYVVVDPTAVKIRAEDRRSVHGV 362
Lm    GLPHGLTLIAGGGFHGKSTLLRALELGVYNHVPDDGRTFVVVDPTAVKIRAEDRRAVHGT 418
                 Q loop motif C          Walker B  D loop
Tc    DISPFINNLPFQKTTTSFVTSDASGSTSQAANIMEALELGSRLLLLDEDTCATNLMYRDD 415
Tb    DISPFINNLPFGKTTNFFVTADASGSTSQAANIMEALELGSQLLLLDEDTCATNLMYRDA 422
Lm    DISPFITNLPYRDNTTAFVTADASGSTSQAANIMEALELGSTTLLLDEDTSATNFMYRDP 478
                             H
Tc    LMQQLVPREQEPITPFVDRVTDLIQNHGVSSIMVIGGSGQYFPHATVTLVMNAYKAFDCS 475
Tb    LMQMLVPRAQEPITPFVERVADLSQNHGVSSIMVIGGSGQYFPQARVVLVMNAYQISDCT 482
Lm    LMEQLVPRTQEPITCFVHRVRDLMYHHGVSVIMVVGGSGQYFPMADVVLLLNAYKVTDAT 538

Tc    ERAKEISR--------TFMSSVQPPKKAISAFVSPMQRQFDGSGTFSTVHCRRGHD---- 523
Tb    KEAKEIASN-------SSLPALNPPGDTASVFIPDVNRCFDPDGSFTTVRRRRGRE---- 531
Lm    AQAKAIAAQSVGRGGVGLVESAAPP--ATSAFRLPPRRSFVYADTFGRLGSQSQQQQYHH 596

Tc    -----------SIKVSGVGVDSIRFAEETIDLSLVEQIVEEGQVNAIAQCLAMLYDGEEN 572
Tb    -----------GTKVSGIGTESIRFSEETIDLSMVEQIVEEGQVNAIAQCLALLYDGEPR 580
Lm    HHGYGYGGSGRGIKISGSGIEHIRIADEDINVTLLEQLVEEGQLNAIAQCLAMLYDSGSA 656

Tc    GIRTILAKGK-------SLKLLYSPSGGSEP-RNVPFYSEFAALIEGCDAALRDARLEAR 624
Tb    IVPEMTTKGG-------ALTQLPSPGGVCEI-QRGKFNSNFSSMIAGCCSHQHDKRLELR 632
Lm    AAAEWQREEAPRSRYPPSLSKALSVDAAAAAGAPASSLSDFGRLVYNCEGRLRQARLELQ 716
```

```
Tc    TPSCYLPRGFTSAARRFEIGAALNRLRTLRTLTAAMK 661
Tb    TPSCYLPRGFTSATRHIEIGAALNRLRTLRTVTAKR- 668
Lm    TASCYLPVGFTALPRVFEIGAALNRLRTLVTSCK--- 750
```

C

```
                                   SAM 2
Tb    MS--SAVDSLLVRLKIPDLIPKFREMNVDRIISLRKLSEEELRTAVPDDDQRNRIIEAIK 58
Tc    MN--SAVDSLLMRLKLSDLIPKFKEMKLDRIVNLRKLSEEELREAVPDDEQRNLIIHAIS 58
Lm    MSQQNAVEALLARLQMGDTIPAFQSMGVDRIVSLRKMSEEALSQAVPDPEQRQALMDAIE 60

Tb    NRGSSE-KRTQAPAASINPPRSTD-DGGRGGMSYPRG--APRGGGPVRGRGRGGTGSAGE 114
Tc    NRGTHE-KRQQSQAANINPPRNAD-DGGRVGMSFPRG--STRGG-MGRGVSRGGRGGGST 113
Lm    SRGHLQSRRAGPPAGQPHPPRSADADFARGGMDEGRGGYAPRGD-RGGRIARGGRGGG-- 117
                                   ZF
Tb    GGFHGSGSNNANMFPSRQRVCNHFLNGD-CRYGDSCRYSHN----KALCQ-EAAESVPRR 168
Tc    N--NNSNSETNTSFPGRQRACNHFFSGE-CKFGDRCRYSHD----KEIYDREMAENGPRR 166
Lm    ---RGAGAPNG---DGAARVCRHVFTPEGCKYGDKCRYSHDENDRRRAQEVPTGSGMAQR 171
                                        KH
Tb    NHDATSLSEDFSETCIIPTHRIKYLLANRADRLRSIHSKNRTHNKSFQHIDPTIEKFELV 228
Tc    PNDSVSKPKDYSEVCEIPTHRIKFLLAKKAERLKQIHIQNHTHNEPFKRVDSNVEKFELV 226
Lm    SLERPPPNFKFSIEVEIPTERIKYLLGSQGNNMKFINETCGTYNERFDHVDEKEETFTIR 231

Tb    IYGADQQSVQESKKMILACIGVTREEEQKNRVQYTMNELSSNQRAAKFLAACNMKNEGTV 288
Tc    VYGPDPQSVLQSKKMILSYVGVTKQEEQKSRLQYTINELSSNQHAAKLLAACNIKNEGTA 286
Lm    ILGGSEEAINKAKEMLLLSAGVKREEEKKDRFQYAVNELDANTHAARLFAACNTKNKDTT 291

Tb    RELSEASLRNIISFFRFEKQQDIRHFWVNTN-SEHSKLDIIANIVAQLQGVQAIMFCDQK 347
Tc    RELSEETLKSIISFFRFEKQQDVRHFYLNVN-HDRVKLDKVAKIVAQLRGVQAIMFSDQK 345
Lm    RHLSDSIMRNIVSSFHFVRPQEVRHFYMYTSSSDKDKLEMVSKIVSQLKGVQAILFCDQK 351
                                 Helicase
Tb    RVVDMSKVASKITRYFNGVSPLFLHRAIPKEERMKMLQTFKDGKPNENGIRERLLVTNED 407
Tc    RVMEMSKVASKIARFFNGVTPLFLHREIPKEERMRILQEFKDGEENESGIRERLLITNED 405
Lm    RVEEMCKGSQRIARHFNGVEPQFVYRQLSKEDRMMALERFKKGVENENGVRQRLLVTNED 411

Tb    YAKLARKTIVPYVNLVINYSVPRSEEYYLLQSLVAGRSDTVGVSIVCVFPHEQSLFQELQ 467
Tc    YAKLARKTLIPYVNLVINYVVPRTEEYYLLQSLVAGRSDTVGVSILCVSPYDQSTFHELQ 465
Lm    YAKLARKTIIPYVNLVINFSVPRAEEYYVLQSQVAGRQGTIGASFLCVSNYEEALFRELE 471

Tb    RNIPFEELEEEGNFKDAAVKLVYDTVDEPLTGEDADPPSNWHEQLKPKTL-------- 517
Tc    QKIVFTAIEEESDFMETAVQLVYDTTANPLTDEDADPPRDWKEKLEPKNASA------ 517
Lm    KNIQFERFEDENHFRTTAVELSYDTTAQPLTPEDADPPADWREHLNPKKPRQNFGKRR 529
```

S2 Fig. Sequence alignments were performed using ClustalW multiple sequence alignment program (http://www.genome.jp/tools/clustalw/). The alignment with the trypanosomatid species; *Trypanosoma brucei* (Tb), *Trypanosoma cruzi* (Tc), and *Leishmanina major* (Lm), are presented. Residues shown in red, blue and green represent identity, similarity and weak similarity, respectively. The sequences were obtained from GeneDB. **(A)** Sequence alignment of *T. brucei* p22 (Tb927.7.7460) with its homologues. The PDIb (protein disulfide isomerase) is indicated. **(B)** Sequence alignment of *T. brucei* p72 ATPase (Tb927.3.1590) with its homologues. **(C)** Sequence alignment of *T. brucei* ZC3H41 (Tb927.11.1980) with its homologues. The different domains are indicated; SAM, sterile alpha motif; ZF, zinc finger; KH, K homology domain.
