# Supplementary material for: Exosome secretion affects social motility in Trypanosoma brucei
Source: PLoS Pathog. 2017 Mar 3;13(3):e1006245. doi: 10.1371/journal.ppat.1006245 (PMC5352147; doi:10.1371/journal.ppat.1006245)
Supplement: S3 Fig — (A) Western analysis. Cells expressing the T7 opposing silencing constructs for SmE/p22 and tagged PTP-p22 construct, or SmE/p72 with the PTP-p72 tagged construct, were silenced for 48 hrs. Cells (~106 cells/ lane) were subjected to western analysis using PTB1 antibodies, which also recognize the tagged protein. (B) The silencing of p22 and p72 affect SL RNP-C stability. Cells carrying the SmE/p22 and SmE/p72 silencing constructs were induced for 48 hrs. RNA (10 μg of total RNA) was subjected to primer extension with primers specific to SL RNA, U4, and U3 snoRNAs (listed in S2 Table). The extension products were separated on a 6% denaturing gel. The identity of the cell line and the position of the modified cap are indicated. The statistical analysis represents the mean ± s.e.m of quantification from three independent experiments. **P <0.01, and ***P <0.005 compared to–Tet, using Student's t-test. (C) Localization of p22 and p72 before and after silencing. Cells expressing PTP-p72 and the SmD1 silencing construct, either un-induced or induced for the indicated times were fixed, and fluorescence was monitored. Nuclei were stained with DAPI. (PDF) [file ppat.1006245.s003.pdf]

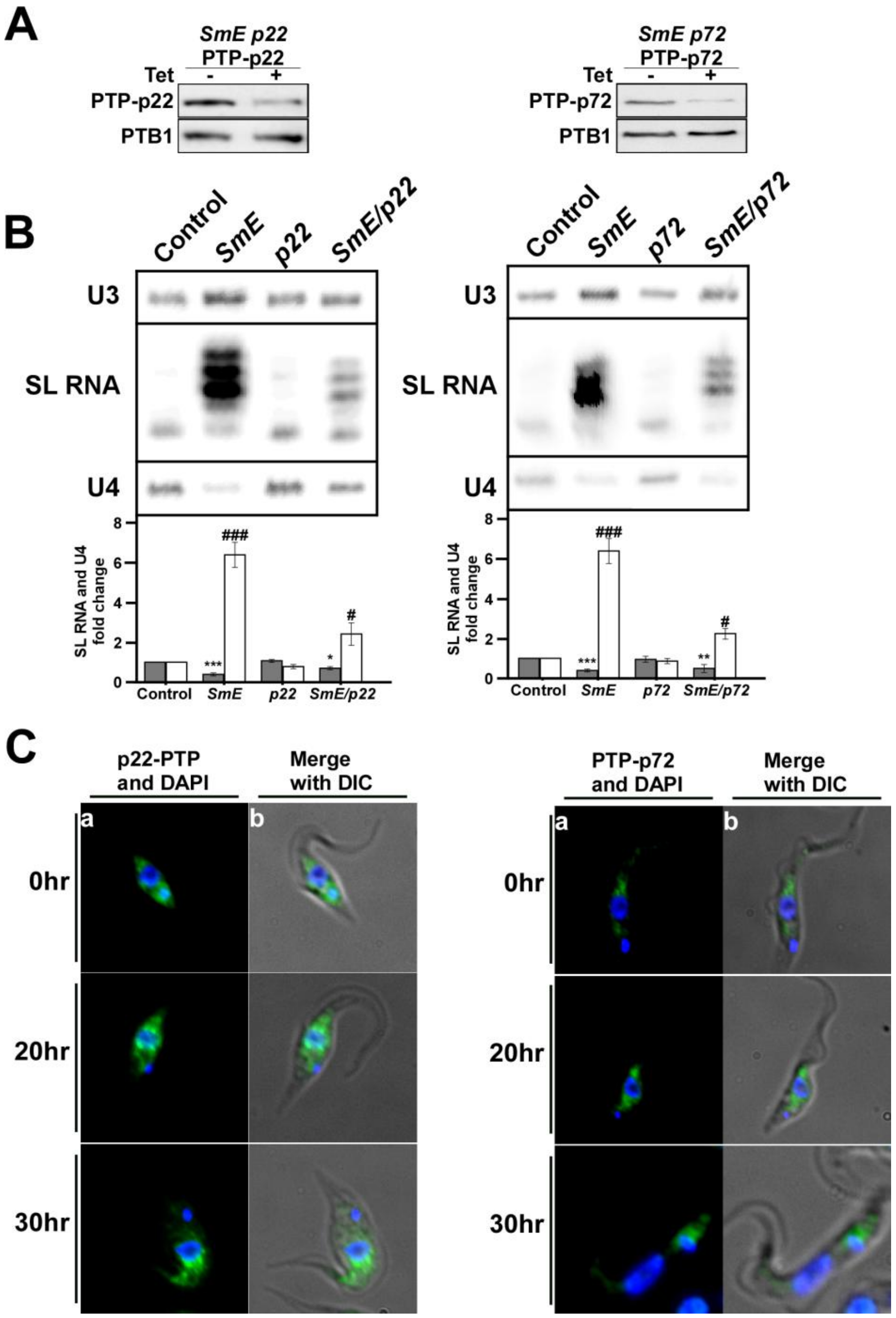

S3 Fig. Co-silencing of *SmE* with either *p22* or *p72*. **(A)** Western analysis. Cells expressing the T7 opposing silencing constructs for *SmE/p22* and tagged PTP-p22 construct, or *SmE/72* with the PTP-p72 tagged construct, were silenced for 48 hrs. Cells (~10^6 cells/ lane) were subjected to western analysis using PTB1 antibodies, which also recognize the tagged protein. **(B)** The silencing of *p22* and *p72* affect SL RNP-C stability. Cells carrying the *SmE/p22* and *SmE/p72* silencing constructs were induced for 48 hrs. RNA (10 µg of total RNA) was subjected to primer extension with primers specific to SL RNA, U4, and U3 snoRNAs (listed in S2 Table). The extension products were separated on a 6% denaturing gel. The identity of the cell line and the

position of the modified cap are indicated. The statistical analysis represents the mean ± s.e.m of quantification from three independent experiments. **P <0.01, and ***P <0.005 compared to –Tet, using Student's *t*-test**.** **(C)** Localization of *p22* and *p72* before and after silencing. Cells expressing PTP-p72 and the *SmD1* silencing construct, either un-induced or induced for the indicated times were fixed, and fluorescence was monitored. Nuclei were stained with DAPI.
