# Supplementary material for: Exosome secretion affects social motility in Trypanosoma brucei
Source: PLoS Pathog. 2017 Mar 3;13(3):e1006245. doi: 10.1371/journal.ppat.1006245 (PMC5352147; doi:10.1371/journal.ppat.1006245)
Supplement: S4 Fig — Cells carrying the SmD1 silencing construct were induced for the times indicated and subjected to in situ hybridization with SL RNA (red), and IFA with ZC3H41 antibodies (green). The nucleus was stained with DAPI. The merge was performed on DAPI staining and SL RNA hybridization. The time points post-silencing are indicated. (PDF) [file ppat.1006245.s004.pdf]

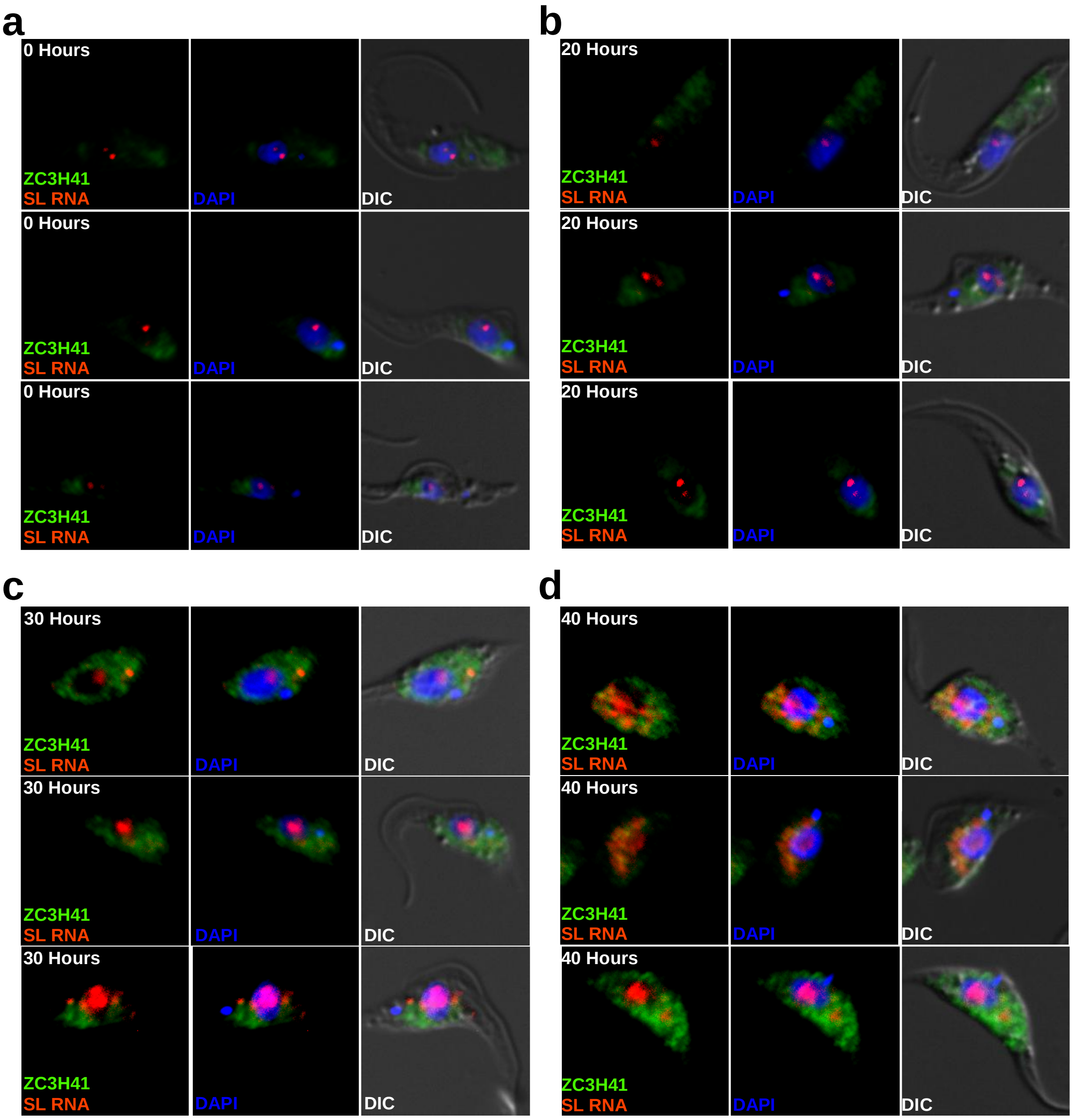

S4 Fig. Changes in localization of ZC3H41 and SL RNA during *SmD1* silencing. Cells carrying the *SmD1* silencing construct were induced for the times indicated and subjected to *in situ* hybridization with SL RNA (red), and IFA with ZC3H41 antibodies (green). The nucleus was stained with DAPI. The merge was performed on DAPI staining and SL RNA hybridization. The time points post-silencing are indicated.
