# Supplementary material for: Exosome secretion affects social motility in Trypanosoma brucei
Source: PLoS Pathog. 2017 Mar 3;13(3):e1006245. doi: 10.1371/journal.ppat.1006245 (PMC5352147; doi:10.1371/journal.ppat.1006245)
Supplement: S5 Fig — (A) Northern blot analysis of cells carrying the silencing construct for SmD1/Mtr4. RNA was prepared from un-induced cells (-Tet) and cells after 2 days of induction (+Tet). Total RNA (20 μg) was subjected to Northern analysis with anti-sense RNA probe to Mtr4 (Tb927.10.7440). The mRNA transcripts, dsRNA, as well as 7SL RNA are indicated. (B) Quantification of changes in SL and U3 snRNA. The ratio between SL RNA and U3 was calculated for each time point that is presented in Fig 2A (SmD1 silenced cells) and in Fig 2B (SmD1/Vps36 silenced cells). (C) As in (B) but showing the ratio between U2 and U3 snRNAs. (D) ZC3H41 is present mostly outside of P-bodies. ZC3H41 localization was determined with respect to P-bodies labeled with DHH1. Cells carrying the SmD1 silencing construct and the YFP-DHH1 construct were silenced for 2 days and subjected to IFA using ZC3H41 and YFP antibodies (red and green, respectively). The nucleus was stained with DAPI. (E) Cytoplasmic SL RNA is not found in P-bodies. Cells carrying the SmD1 silencing construct and expressing YFP-DHH1 were induced for 2 days and subjected to in situ hybridization with SL RNA (red), and immunofluorescence using YFP antibody for YFP-DHH1 (green). The nucleus was stained with DAPI. (F) SL RNA granules are distinct from stress granules. Cells were silenced for 2 days and stained by IFA using PTB1 antibodies (green stain) and subjected to in situ hybridization with SL RNA (red). The nucleus was stained with DAPI. (G) As in F but using antibodies to eIF4E-1. The merge was performed between DAPI staining, IFA and in situ hybridization. (PDF) [file ppat.1006245.s005.pdf]

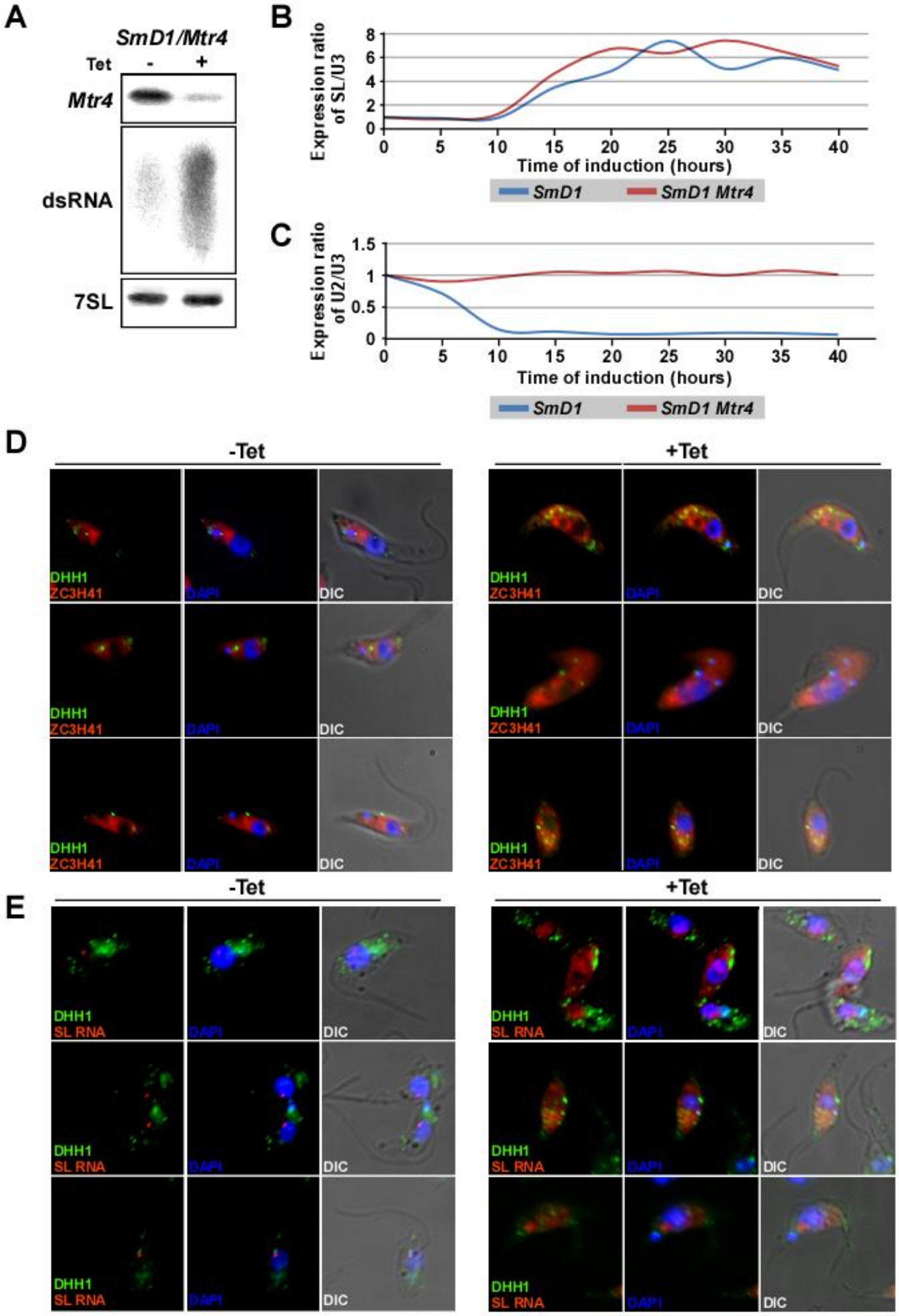
A
SmD1/Mtr4
Tet - +
Mtr4
dsRNA
7SL
B
Expression ratio of SL/U3
Time of induction (hours)
SmD1
SmD1 Mtr4
C
Expression ratio of U2/U3
Time of induction (hours)
SmD1
SmD1 Mtr4
D
-Tet
+Tet
DHH1
ZC3H41
DAPI
DIC
E
-Tet
+Tet
DHH1
SL RNA
DAPI
DIC

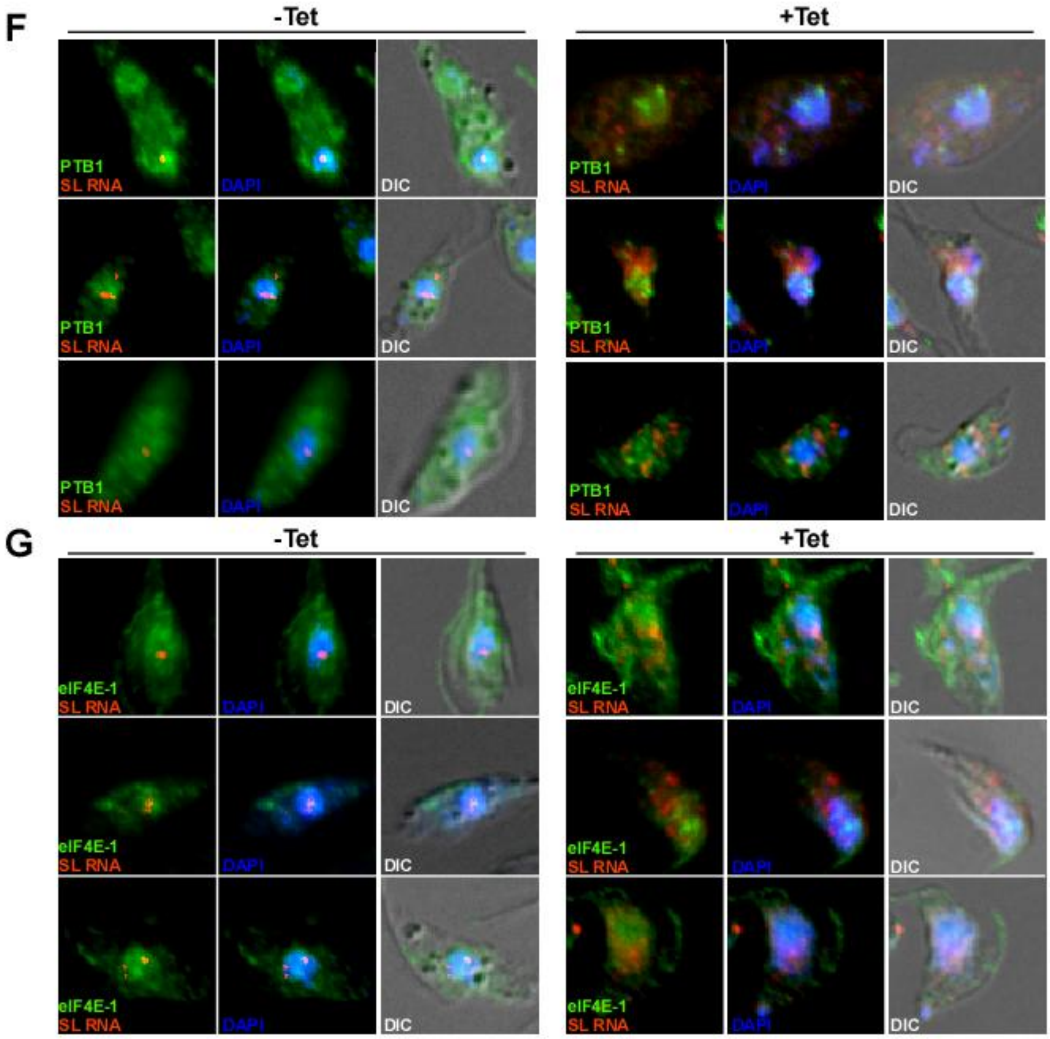


**S5 Fig. MTR4 silencing (A)** Northern blot analysis of cells carrying the silencing construct for *SmD1/Mtr4*. RNA was prepared from un-induced cells (-Tet) and cells after 2 days of induction (+Tet). Total RNA (20 µg) was subjected to Northern analysis with anti-sense RNA probe to *Mtr4* (Tb927.10.7440). The mRNA transcripts, dsRNA, as well as 7SL RNA are indicated. **(B)** Quantification of changes in SL and U3 snRNA. The ratio between SL RNA and U3 was calculated for each time point that is presented in Figure 2A (*SmD1* silenced cells) and in Figure 2B (*SmD1/VPS36* silenced cells). **(C)** As in **(B)** but showing the ratio between U3 and U3 snRNAs. **(D)** ZC3H41 is present mostly outside of P-bodies. ZC3H41 localization was determined with respect to P-bodies labeled with DHH1. Cells carrying the *SmD1* silencing construct and the YFP-DHH1 construct were silenced for 2 days and subjected to IFA using ZC3H41 and YFP antibodies (red and green, respectively). The nucleus was stained with DAPI. **(E)** Cytoplasmic SL RNA is not found in P-bodies. Cells carrying

the *SmD1* silencing construct and expressing YFP-DHH1 were induced for 2 days and subjected to *in situ* hybridization with SL RNA (red), and immunofluorescence using YFP antibody for YFP-DHH1 (green). The nucleus was stained with DAPI. **(F)** SL RNA granules are distinct from stress granules. Cells were silenced for 2 days and stained by IFA using PTB1 antibodies (green stain) and subjected to *in situ* hybridization with SL RNA (red). The nucleus was stained with DAPI. **(G)** As in **F** but using antibodies to eIF4E-1. The merge was performed between DAPI staining, IFA and *in situ* hybridization.
