# Supplementary material for: Exosome secretion affects social motility in Trypanosoma brucei
Source: PLoS Pathog. 2017 Mar 3;13(3):e1006245. doi: 10.1371/journal.ppat.1006245 (PMC5352147; doi:10.1371/journal.ppat.1006245)
Supplement: S6 Fig — Cells were fixed after 2 days of silencing, and ultra-thin sections were prepared. The different ultra-structures are indicated. M, mitochondrion; ER, enodoplasmic reticulum; A, double-membrane autophagosome; Scale bars are indicated. (PDF) [file ppat.1006245.s006.pdf]

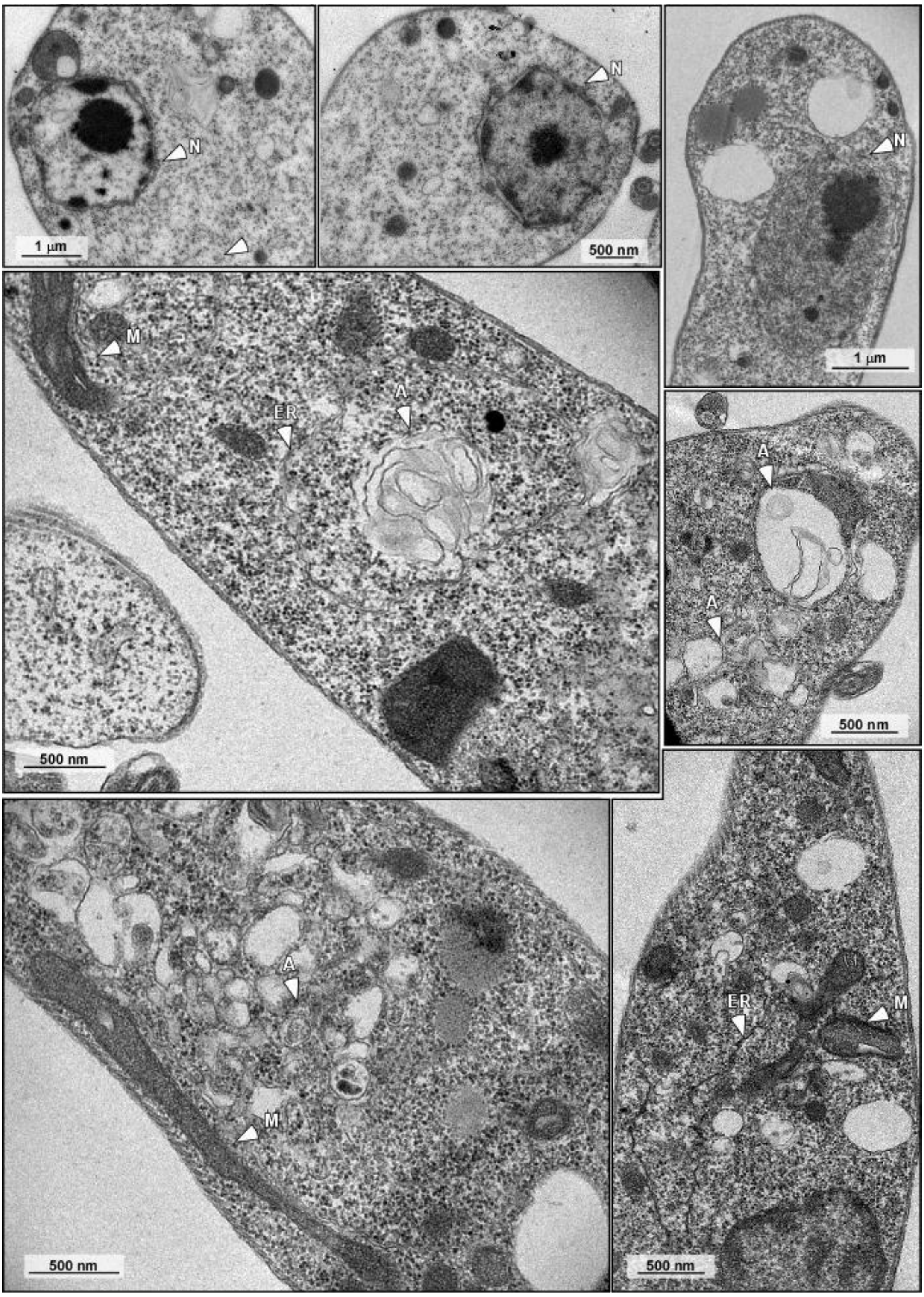

S6 Fig. TEM of SEC63 silenced cells. Cells were fixed after 2 days of silencing, and ultra-thin sections were prepared. The different ultra-structures are indicated. M, mitochondrion; ER, enodoplasmic reticulum; A, double-membrane autophagosome; Scale bars are indicated.
