# Supplementary material for: Exosome secretion affects social motility in Trypanosoma brucei
Source: PLoS Pathog. 2017 Mar 3;13(3):e1006245. doi: 10.1371/journal.ppat.1006245 (PMC5352147; doi:10.1371/journal.ppat.1006245)
Supplement: S7 Fig — Cells carrying the SEC63 construct were silenced for 2 days and then fixed and visualized under EM. The scale bar is indicated. Exosomes are marked with arrowheads. (PDF) [file ppat.1006245.s007.pdf]

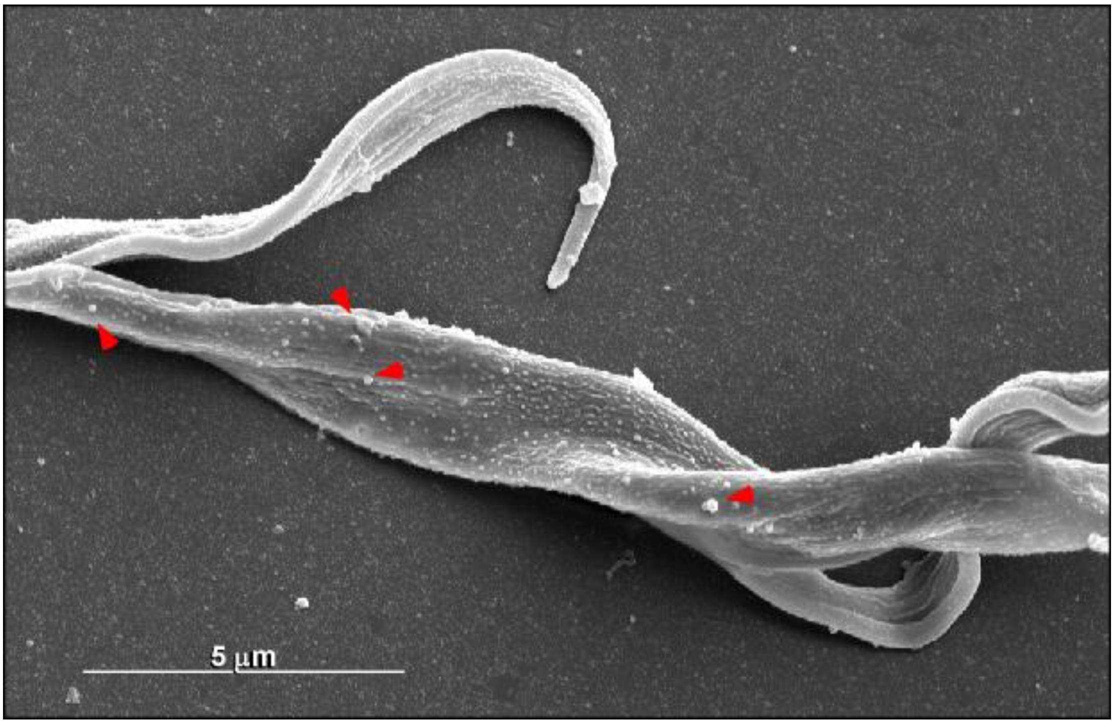

S7 Fig. Exosome detection by SEM of *SEC63* silenced cells. Cells carrying the SEC63 construct were silenced for 2 days and then fixed and visualized under EM. The scale bar is indicated. Exosomes are marked with arrowheads.
