# Supplementary material for: Exosome secretion affects social motility in Trypanosoma brucei
Source: PLoS Pathog. 2017 Mar 3;13(3):e1006245. doi: 10.1371/journal.ppat.1006245 (PMC5352147; doi:10.1371/journal.ppat.1006245)
Supplement: S8 Fig — (A) Western analysis demonstrating the depletion of Vps36. Cells carrying the SmD1 silencing construct and the PTP-Vps36 tagging, un-induced (-Tet) and 2 days after induction (+Tet) were subjected to western analysis. PTB1 was used to control for equal loading. (B) Northern analysis demonstrating the silencing of Vps36. RNA was prepared from un-induced cells (-Tet) and cells after 2 days of induction (+Tet). Total RNA (20 μg) was subjected to Northern analysis with anti-sense RNA probe to Vps36. The mRNA transcripts, dsRNA, as well as 7SL RNA are indicated. (C) SL RNA was elevated in cells silenced for SmD1/Vps36. Cells carrying the silencing constructs were induced for the times indicated. RNA was subjected to primer extension with SL RNA and U3 probes. The position of the cap nt is indicated. (D) Growth curves of SmD1, and SmD1/Vps36 silenced cells. The identity of the cell lines and treatment are indicated. (PDF) [file ppat.1006245.s008.pdf]

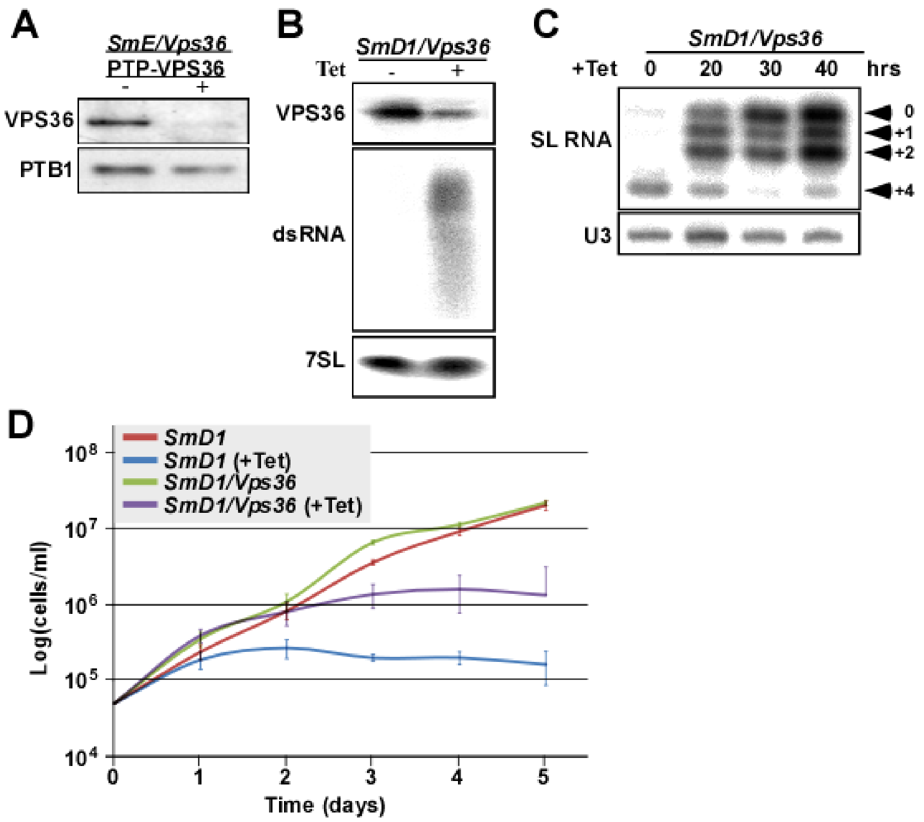

S8 Fig. Silencing of *Vps36* does not affect the accumulation of SL RNA; inhibition of growth induced by *SmD1* silencing. **(A)** Western analysis demonstrating the depletion of Vps36. Cells carrying the *SmD1* silencing construct and the PTP-Vps36 tagging, un-induced (-Tet) and 2 days after induction (+Tet) were subjected to western analysis. PTB1 was used to control for equal loading. **(B)** Northern analysis demonstrating the silencing of *Vps36*. RNA was prepared from un-induced cells (-Tet) and cells after 2 days of induction (+Tet). Total RNA (20 µg) was subjected to Northern analysis with anti-sense RNA probe to Vps36. The mRNA transcripts, dsRNA, as well as 7SL RNA are indicated. **(C)** SL RNA was elevated in cells silenced for *SmD1/Vps36*. Cells carrying the silencing constructs were induced for the times indicated. RNA was subjected to primer extension with SL RNA and U3 probes. The position of the cap nt is indicated. **(D)** Growth curves of *SmD1*, and *SmD1/Vps36* silenced cells. The identity of the cell lines and treatment are indicated.
