# Supplementary material for: Exosome secretion affects social motility in Trypanosoma brucei
Source: PLoS Pathog. 2017 Mar 3;13(3):e1006245. doi: 10.1371/journal.ppat.1006245 (PMC5352147; doi:10.1371/journal.ppat.1006245)
Supplement: S9 Fig — Cells were subjected to heat shock (37°C for 40 min) and then returned to 26°C; growth was monitored in comparison to cells which were not subjected to heat-shock. (PDF) [file ppat.1006245.s009.pdf]

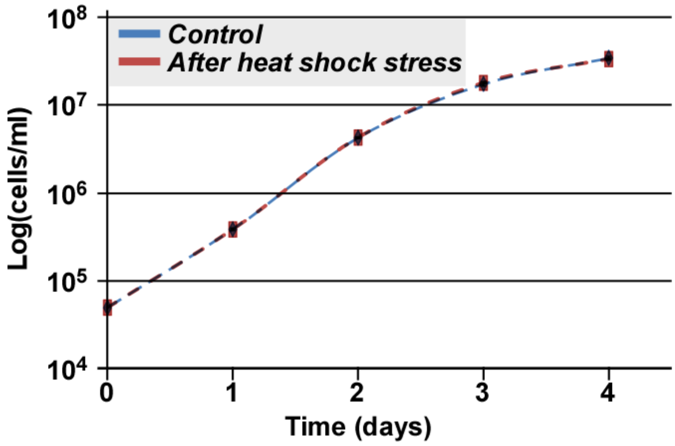

S9 Fig. Cells continue to grow normally after heat shock. Cells were subjected to heat shock (37°C for 40 min) and then returned to 26°C; growth was monitored in comparison to cells which were not subjected to heat-shock.
