# Supplementary material for: Exosome secretion affects social motility in Trypanosoma brucei
Source: PLoS Pathog. 2017 Mar 3;13(3):e1006245. doi: 10.1371/journal.ppat.1006245 (PMC5352147; doi:10.1371/journal.ppat.1006245)
Supplement: S10 Fig — Exosomes were prepared from SmD1 silenced cells (109) after 2 days of silencing. The exosomes were treated with 0.05% NP40 for one hour and then analyzed by NanoSight instrument. Untreated exosomes (red), and treated exosomes (blue). (PDF) [file ppat.1006245.s010.pdf]

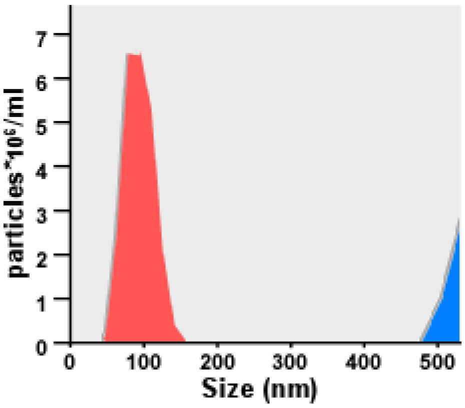

S10 Fig. NanoSight analysis. Exosomes were prepared from *SmD1* silenced cells (10^9) after 2 days of silencing. The exosomes were treated with 0.05% NP40 for one hour and then analyzed by NanoSight instrument. Untreated exosomes (red), and treated exosomes (blue).
