# Supplementary material for: Exosome secretion affects social motility in Trypanosoma brucei
Source: PLoS Pathog. 2017 Mar 3;13(3):e1006245. doi: 10.1371/journal.ppat.1006245 (PMC5352147; doi:10.1371/journal.ppat.1006245)
Supplement: S1 Table — (PDF) [file ppat.1006245.s011.pdf]

Supplementary Table 1: The oligonucleotides used are listed blow.

Molecule	Restriction Site	Sequence	Orientation	Site in the gene	Insert size (bp)
**Oligonucleotide for Affinity Selection of SL RNP-C**					
SL RNA		5'-GGAGCUUCUCAUACXXXXA-3' (X denotes a biotinylated 2'-deoxycytidine)	antisense	40-54	
		Oligonucleotides for T7 Opposing Silencing Constructs			
SmE	XhoI	5'-CCG CTC GAG ATG AGC GTC ACA ACA AAG CA-3'	sense	20-ינו	380
SmE	HindIII	5'-CCC AAG CTT TAT GCC AAT AGG GTG AAT GAC-3'	antisense	237-258	380
ZC3H41-p58	XhoI	5'-CCG CTC GAG TAA TGG TGA CTG CAG GTA CGG-3'	sense	416-437	518
ZC3H41-p58	HindIII	5'-CCC AAG CTT CAA AGC GGA AGA ACG AAA TG-3'	antisense	896-916	518
p72	XhoI	5'-CCG CTC GAG TCC ACA GGG TTC TCA CCA C-3'	sense	141-160	489
p72	HindIII	5'-CCC AAG CTT ATG CCC ATC AAT ACG TCG -3'	antisense	594-612	489
p22	XhoI	5'-CCG CTC GAG TGA GCA GCA CAG TTA GGC -3'	sense	242 – 259	237
p22	HindIII	5'-CCC AAG CTT AGT TCT GTA TCT TCC TCA TTG -3'	antisense	440 – 461	237
NMP	XhoI	5'-CCG CTC GAG ATG GCG ACG GAC GAA CGA TT-3'	sense	20-ינו	533
NMP	HindIII	5'-CCC AAG CTT CCC CTT CAT CCC CAC CCG-3'	antisense	520-540	533
VPS36	XhoI	5'-TTT CTC GAG ATT TGA ACT ATG ACG AAG GC-3'	sense	101-120	550
VPS36	HindIII	5'-TTT AAG CTT GCG ATA TTT TGC TGC TTT TG-3'	antisense	631-650	550
Vps24	XhoI	5'-TTT CTC GAG CAA GCG CTG TTG TTA TGA CA-3'	sense		
Vps24	HindIII	5'-TTT AAG CTT TCA ACT AAC AGT CCC ACG AA-3'	antisense		
**Oligonucleotides for T7 Opposing Silencing Constructs**					
ZC3H41-p58	XbaI	5'-GTA CTG TCT AGA ATA AAT CCA CCT CGT TCT ACG G-3'	sense	220-241	503
ZC3H41-p58	MluI	5'-TCG TAC ACG CGT GCT CTC CTG AAC AGA TTG CTG G-3'	antisense	699-721	503
p58	HindIII	5'-TCG TAC AAG CTT GCT CTC CTG AAC AGA TTG CTG G-3'	antisense	699-723	503
Mtr4	XbaI	5'-CCC TCT AGA AAG CGA GTT ATT TAC ACT TCA-3'	sense		
Mtr4	MluI	5'-CCC ACG CGT TAG CAC GCC TGA AAT TGT-3'	antisense		
Mtr4	HindIII	5'-CCC AAG CTT TAG CAC GCC TGA AAT TGT-3'	antisense		
SmD1	XbaI	5'-GCT CTA GAA TGC CCG CGG CGG AGT CAT-3'	sense	304-323	317
SmD1	MluI	5'-CGA CGC GTC TCA TTT GAT CGC TCC GTC CT-3'	antisense	601-621	317
SmD1	HindIII	5'-CCA AGC TTC TCA TTT GAT CGC TCC GTC CT-3'	antisense	601-621	317
**Oligonucleotides for YFP/RFP tagging**					
RAB28	HindIII	5'-CCC AAG CTT CCA TGA GTA GCG ACA GTT CAG A-3'	sense		
RAB28	NotI	5'-CCC GCG GCC GCC ATC ACT GCG CAT TTA CCC T-3'	antisense		
VPS36	HindIII	5'-TTT AAG CTT CCA TGA GTT CAT GGG AAT CG-3'	sense		
VPS36	NotI	5'-TTT GCG GCC GCT GAT GCC CGC ATG AGA CC-3'	antisense		
VPS28	HindIII	5'-CCC AAG CTT CCA TGG AAG TGG CAT TCA CCA T-3'	sense		
VPS28	NotI	5'-CCC GCG GCC GCT TAC GCC TTT GGC CGT CGC G-3'	antisense		
**Oligonucleotides for antibody for p58**					
ZC3H41-p58	EcoRI	5'-CCC GAA TTC ATG AGC AGC GCG GTA GAT-3'	sense		
ZC3H41-p58	HindIII	5'-CCC AAG CTT CTA ACT GAA ATC CTC AGA CAA C-3'	antisense		
**Oligonucleotides for PTP Tagging**					
NMP	HindIII	5'-AAA AAG CTT GGA TGG CGA CGG ACG AAC GAT T-3'	sense		
NMP	ApaI	5'-TTT GGG CCC TGG TCG TAC ATC ATT TTT TC-3'	antisense		
p22	ApaI	5'-AAA GGG CCC TTA CGC GGT TGT GCA CTG-3'	sense		
p22	NotI	5'-AAA GCG GCC GCC CCC TCC TCA CCG TTG GTT T-3'	antisense		
P72	HindIII	5'-AAA AAG CTT GGA TGT ACC ATC GTG GCT ACG-3'	sense		
P72	ApaI	5'-TTT GGG CCC CTC GAT TTC TGC CTG CAT AC-3'	antisense		
Vps36	HindIII	5'-AAA AAG CTT GGA TGA GTT CAT GGG AAT CGT G-3'	sense		
Vps36	ApaI	5'-TTT GGG CCC ACA TTT CAC TGT GTC GGA GC-3'	antisense		
**Oligonucleotides for Primer Extension**					
TbSL RNA		5'-GGG AGC TTC TCA TAC-3'	antisense	40-54	
TbU3		5'-TGC CGT TCA TCG AAC-3'	antisense	107-121	
TbU4		5'-CAA ACT TTC CCC GAA GGA-3'	antisense	76-93	
**Oligonucleotides for Northern**					
R-VPS36-T7		5'-TTA ATA CGA CTC ACT ATA GGG AGA CTC TTT GAT GTT GCC TAC TT-3'	antisense		
antisense T7-Mtr4		5'-TTA ATA CGA CTC ACT ATA GGG AGA TAG CAC GCC TGA AAT TGT-3'	antisense		
