# Supplementary material for: Exosome secretion affects social motility in Trypanosoma brucei
Source: PLoS Pathog. 2017 Mar 3;13(3):e1006245. doi: 10.1371/journal.ppat.1006245 (PMC5352147; doi:10.1371/journal.ppat.1006245)
Supplement: S2 Table — The list includes data from three independent purifications. The mass spectrometry data were analyzed using the Sequest 3.31 software (J. Eng and J.Yates, University of Washington and Finnigan, San Jose) searching against T. brucei TriTryp database version 5 (http://tritrypdb.org/tritrypdb/). To control for the selective affinity selection (-Oligo). The affinity selection protocol was performed using fractions containing the SL RNA complex purified on a FPLC column but in the absence of oligonucleotide. The proteins associated with the beads were separated on a gel for a short time capturing the entire protein population. The gel slice was analyzed by MS. (PDF) [file ppat.1006245.s012.pdf]

Supplementary Table 2: The list of proteins identified by Mass-spectrometry. The list includes data from three independent purifications. The mass spectrometry data were analyzed using the Sequest 3.31 software (J. Eng and J.Yates, University of Washington and Finnigan, San Jose) searching against *T. brucei* TriTryp database version 5 (http:// tritrypdb.org/tritrypdb/). To control for the selective affinity selection (-Oligo). The affinity selection protocol was performed using fractions containing the SL RNA complex purified on a FPLC column but in the absence of oligonucleotide. The proteins associated with the beads were separated on a gel for a short time capturing the entire protein population. The gel slice was analyzed by MS.

experiment Number	Identified Protein Annotation	Peptide	Peptide Repeat		Accession no.	Molecular Weight (kDa)
			+ Oligo	- Oligo		
experiment 1	50S ribosomal protein L7Ae, putative	K.ITSIKAK.H	2		Tb927.4.750	16.30
experiment 1	40S ribosomal protein S6, putative	-.MKLNVAYPRNGTVK.Q	3		Tb927.10.190	28.40
experiment 1	zinc finger protein family member, putative (ZC3H41)	K.SFQHIDPTIEK.F<br>R.LLVTNEDYAK.L	2		Tb927.11.1980	58.40
experiment 1	hypothetical protein, conserved	K.SRIPLDK.F	2		Tb927.8.560	64.60
experiment 1	serine/threonine protein kinase, putative	R.LAYSLSR.N	3		Tb927.11.6990	67.10
experiment 1	fatty acyl CoA syntetase 1 (ACS1)	R.IFETIRKVVESR.L	6		Tb927.9.4190	78.90
experiment 1	hypothetical protein, conserved	R.IAELEEKRR.A	4		Tb927.8.3840	80.00
experiment 1	hypothetical protein, conserved	K.TAPQEHEKKAVDDFPDGATR.G	4		Tb927.9.4350	94.10
experiment 1	ubiquitin-activating enzyme E1, putative (UBA1)	R.IDINEGRDITVK.E	4		Tb927.8.2640	116.40
experiment 1	hypothetical protein, conserved	R.LLEETEQEFAPAPYVR.G	2		Tb927.8.4230	119.00
experiment 1	Protein of unknown function, putative	R.QEYNKNTQR.G<br>R.ETPEGEDEK.F<br>R.QEYNKNTQR.G	10		Tb927.5.3290	130.00
experiment 1	Clustered mitochondria, putative	K.GAVAIEKEMLQRVK.D	3		Tb927.8.4780	468.10
experiment 2	mitochondrial RNA binding complex 1 subunit (MRB1590)	K.VSGIGTESIR.F<br>K.VLQTTDLAAANPCR.R<br>R.TYVVVDPTAVK.I<br>R.AQEPITPFVER.V<br>R.CFDPDGSFTTVR.R<br>R.SLTNETYQISENVR.C<br>R.VAAEDFILR.S<br>K.GGALTQLPSPGGVCEIQR.G<br>K.EIASNSSLPALNPPGDTASVFIPDVNR.[illegible]	17		Tb927.3.1590	72.60
experiment 2	Polyadenylate-binding protein 2 (Poly(A)-binding protein 2) (Poly(A)-binding protein II) (PABII) (Polyadenylate-binding nuclear protein 1) (Nuclear poly(A)-binding protein 1) (PABP2), putative (PABP2)	R.LTAIGLATDEK.G	2		Tb927.9.10770	62.10
experiment 2	hypothetical protein, conserved	K.YAEVSK.D<br>R.NEEDTELVHK.I<br>R.VVYNTANVDR.S<br>K.SSEEEDIEFDYPEAVLESLAQWTLK.Y	7		Tb927.7.7460	22.30
experiment 3	Protein of unknown function, putative	QEYNKNTQR	1		Tb927.5.3290	130.00
experiment 3	hypothetical protein, conserved	EGLTRGQQAVEVELQGVKESLQLLQK	6		Tb927.5.1180	99.10
experiment 3	hypothetical protein, conserved	RLCLKPFVCQR	3		Tb927.11.16330	103.60
experiment 3	zinc finger protein family member, putative (ZC3H41)	R.NIPFEELEEEGNFKDAAVK.L<br>SEEYYLLQSLVAGR	8		Tb927.11.1980	58.40
experiment 3	Nin one binding (NOB1) Zn-ribbon like, putative	GTNRCLVLR	3		Tb927.11.10860	47.30

experiment Number	Identified Protein Annotation	Peptide	Peptide Repeat		Accession no.	Molecular Weight (kDa)
			+ Oligo	- Oligo		
negative control	phosphoglycerate kinase phosphoglycerate kinase	SGETEDTYIADLVVALGSGQIK		18	Tb927.10.2890	46.56
negative control	beta tubulin beta tubulin	TIGVEDDAFNTFFSETGAGK		12	Tb927.1.2340	49.76
negative control	alpha tubulin alpha tubulin	NDKLYITLQVASAK		5	Tb927.9.10230	18.76
negative control	GTP-binding nuclear protein rtb2, putative GTP-binding nuclear protein rtb2, putative	REIVVQAGQGNQIGSK		12	Tb927.1.2330	49.67
negative control	succinyl-CoA synthetase alpha subunit, putative succinyl-CoA synthetase alpha subunit, putative	EIQVALFSATPDDVLELTKK		7	Tb927.9.4680	45.33
negative control	amidinotransferase, putative amidinotransferase, putative	DVNGFLVGGASLKPEFVDIIK		7	Tb927.11.5520	26.80
negative control	p25-alpha, putative p25-alpha, putative	SINPDEAVAYGAAVQAFILTGGK		18	Tb927.11.11330	71.36
negative control	heat shock 70 kDa protein, mitochondrial precursor, putative heat shock 70 kDa protein, mitoch	ALIGVVSNPVNSVVPIASEVLKK		5	Tb927.10.2560	33.17
negative control	paraflagellar rod component, putative paraflagellar rod component, putative	KAGSTYLNLPVFGTVAEAVK		7	Tb927.3.2230	31.44
negative control	glyceraldehyde 3-phosphate dehydrogenase, glycosomal glyceraldehyde 3-phosphate dehydrogenase,	GVNPDEAVALGAATLGGVLR		10	Tb927.6.3740	71.43
negative control	S-adenosylmethionine synthetase, putative S-adenosylmethionine synthetase, putative	AFQTNAAAFVK		2	Tb927.8.6750	19.25
negative control	heat shock 70 kDa protein, putative heat shock 70 kDa protein, putative	IGGIGTVPVGR		3	Tb927.10.2090	49.07
negative control	Nitroreductase family, putative Nitroreductase family, putative	FFADKPDEATLSPEMKEHYEKFEK		4	Tb927.9.13785	11.07
negative control	Thioredoxin-like, putative Thioredoxin-like, putative	AFNGQPGHLSR		2	Tb927.10.6070	14.56
negative control	glycerol-3-phosphate dehydrogenase [NAD+], glycosomal glycerol-3-phosphate dehydrogenase [NAD+]	GAVFGSGAFGTALAVLAK		6	Tb927.8.3530	37.78
negative control	2-amino-3-ketobutyrate coenzyme A ligase, putative 2-amino-3-ketobutyrate coenzyme A ligase,	SVGSLVSVPVIHGEVLPLPGELGVIEAAK		4	Tb927.4.1300	41.99
negative control	translationally controlled tumor protein (TCTP), putative translationally controlled tumor	LGFLTFPSNLGTTIR		6	Tb927.9.6250	41.57
negative control	Eukaryotic initiation factor 4A-1 Eukaryotic initiation factor 4A-1	KLNNIHTILPVLNHVVR		8	Tb927.10.6400	59.47
negative control	arginine kinase arginine kinase	AVGVIPSTQGK		5	Tb927.6.4280	39.02
negative control	Double RNA binding domain protein 3 Double RNA binding domain protein 3	SINPDEAVAYGAAVQAHIVSGGK		2	Tb927.7.710	70.17
negative control	Co-chaperone protein P23 Co-chaperone protein P23	VLLGATNPADSLPGTIR		2	Tb927.11.16130	16.85
negative control	kinetoplastid membrane protein 11-5 kinetoplastid membrane protein 11-5	ILGDFGVPTR		2	Tb927.10.1400	23.35
negative control	hypoxanthine-guanine phosphoribosyltransferase hypoxanthine-guanine phosphoribosyltransferase	AVILFGEQHTK		2	Tb927.7.2980	21.23
negative control	elongation factor 1-alpha elongation factor 1-alpha	VDFNVPLDDGNITNDYR		3	Tb927.1.710	45.22
negative control	mitochondrial malate dehydrogenase mitochondrial malate dehydrogenase	GKLTIPANVNDSVTK		4	Tb927.11.9590	48.42
negative control	enolase enolase	SREPSYFVHSK		2	Tb927.6.4140	13.00
negative control	DHH1 DHH1	LLGVTLLDGLR		4	Tb927.10.15410	33.69
negative control	universal minicircle sequence binding protein 2 universal minicircle sequence binding protein 2	VDTQLPHIQALLVPTR		4	Tb927.10.3990	46.44
negative control	universal minicircle sequence binding protein 1 universal minicircle sequence binding protein 1	IATGVDGLVR		2	Tb927.11.1890	36.00
negative control	chaperonin HSP60, mitochondrial precursor chaperonin HSP60, mitochondrial precursor	GLQYYDISAK		2	Tb927.3.1120	24.39
negative control	succinyl-CoA ligase [GDP-forming] beta-chain, putative succinyl-CoA ligase [GDP-forming] beta-ch	FVIGGPHGDAGLTGR		2	Tb927.6.4840	43.51
negative control	heat shock protein, putative heat shock protein, putative	GVVFVAISNDADKAK		3	Tb927.7.4520	22.30
negative control	paraflagellar rod component, putative paraflagellar rod component, putative	LNDVIPNATTTSR		2	Tb927.10.11300	14.32
negative control	glycosomal malate dehydrogenase glycosomal malate dehydrogenase	AAENAAEQIK		2	Tb927.10.7410	44.88
negative control	Serine-threonine kinase receptor-associated protein, putative Serine-threonine kinase receptor-	HIDTAAIYKNEESAGR		4	Tb927.11.4700	30.97
negative control	prostaglandin f synthase prostaglandin f synthase	YQSLTNQSVLGDEPHLR		3	Tb927.10.10890	80.71
negative control	triosephosphate isomerase triosephosphate isomerase	EPNAPADAAAGGAAAGGGR		4	Tb927.10.6060	21.82
negative control	S-adenosylhomocysteine hydrolase, putative S-adenosylhomocysteine hydrolase, putative	NAGSLSGVVDR		3	Tb927.4.2740	16.32
negative control	heat shock protein 70 heat shock protein 70	NITPNVTQLSIQNLVQNFGNIK		5	Tb927.9.8740	36.96
negative control	phosphomannose isomerase, putative phosphomannose isomerase, putative	HAEALQEHAEELR		2	Tb927.11.14780	46.45
negative control	nucleoside diphosphate kinase nucleoside diphosphate kinase	EAGTYKVER		2	Tb927.8.6060	43.71
